# The design and development of technology platforms in a developing country healthcare context from an ecosystem perspective

**DOI:** 10.1186/s12911-020-1028-0

**Published:** 2020-03-12

**Authors:** Hilde Herman, Sara S. Grobbelaar, Calie Pistorius

**Affiliations:** 1 Department of Industrial Engineering, Stellenbosch University, New Industrial Engineering Building; Reception on 2nd floor, Banghoek Road, Stellenbosch, 7600 South Africa; 2 DST-NRF Centre of Excellence in Scientometrics and Science, Technology and Innovation Policy (SciSTIP), Stellenbosch University, Stellenbosch, 7600 South Africa

**Keywords:** Technology platforms, Ecosystems, Public health, Healthcare benefits, Developers, Platform owners, Developing countries

## Abstract

**Background:**

Research on the development and functioning of technology platforms specifically for health applications in sub-Saharan Africa (SSA), is limited. The healthcare sector has also been resistant to platform adoption due to characteristics such as sensitive data and high cost of failure. A framework for the design, development and implementation of technology platforms in the South African health context could therefore contribute to the gap in research as well as provide a practical tool that platform owners could use to potentially increase the adoption of platforms in this context.

**Methods:**

The research design for this study was based on the Grounded Theory Conceptual Framework Analysis process. The process focused on mapping and investigating data sources, categorising and integrating concepts, synthesising these concepts into a framework and iteratively evaluating the framework. The first stage of the evaluation process was a preliminary evaluation exploring an existing Health platform in South Africa (MomConnect). The second evaluation stage included local and international interviews with nine experts to identify any missing concepts in the framework. Stage three included a case study and case study interviews which led to the formulation of the final framework and management tool.

**Results:**

The developed and evaluated framework comprised three components, namely the pre-use component, which includes considerations the platform owner should be aware of prior to using the framework. The framework comprises of two dimensions, 1) an ecosystem dimension to guide the platform owner to consider different ecosystem actors before embarking on designing a platform 2) a platform development dimension that include typical platform development components and presents an interpretation of the viewpoints included in the ecosystem levels.

**Conclusions:**

The final framework can be used by platform owners as a management tool. A unique contribution of this study is that the framework draws from two platform perspectives, namely the engineering and the economic perspectives to provide a holistic understanding of platforms. Finally, a contribution of this article is the tailoring of the framework for the South African health context.

## Background

### Introduction

Health systems in sub-Saharan Africa (SSA) are in dire need of effective and sustainable solutions [1]. Initiatives such as the Sustainable Development Goals aim to enhance health and well-being, particularly in developing countries [2]. Low life expectancy, high maternal and neonatal mortality, the impact of HIV and increasing prevalence of non-communicable diseases, for example, are all compounded by a range of challenges, including the limited availability of skilled healthcare professionals [3].

Technology platforms refer to the technology that provide a software-base upon which interactions and transactions between several actors can take place [4] and can be applied in healthcare to promote communication, data analysis and to create an opportunity to advance patient healthcare education [5]. Platform-enabled solutions have significant potential to contribute to the alleviation of health-related challenges in SSA. They offer the ability to improve the quality of and accessibility to healthcare, especially in rural areas of developing countries. Current barriers such as data collection, decision support, remote monitoring, the ability to rapidly obtain and communicate information and patient education can all be addressed by the use of technology platforms, which will in turn lead to increased efficiency and point of care services [6–10].

The accessibility of large volumes of health-related data obtained from sources such as Electronic Health Records (EHRs), data banks, Internet of Things (IoT) sensors, other data-obtaining medical devices and mHealth applications [11], can be utilised through technology platforms and thereby aid in improving quality and accessibility of healthcare. One example may be to increase visibility of medicines throughout the supply chain through a common technology platform accessible by value chain stakeholders intended to improve supply chain resilience and reduce stock-outs.

Building on this premise, the purpose of this research was to develop a framework that can serve as a guide to support the design, development and implementation of technology platforms in the SSA health context and can thereby ultimately contribute to improved experiences for the end-users of health services. It is envisaged that the proposed framework could contribute to the increased and sustained adoption and use of health platforms. By adopting an ecosystem perspective, the proposed framework provides a useful perspective of the platform actors and the environment they encounter in the SSA context. Often ICT4D interventions in healthcare fail due to a lack of uptake, lack of interoperability and coordination among ecosystem participants. For example, end-to-end visibility in the medicine supply chain in South Africa’s (SA’s) public health sector remains an elusive goal with a wide range of participants with different systems and reporting processes – here there remains a lack of a common platform used by all participants [12–15].

The core issue that this study aimed to address was: *How can the design, development and implementation of technology platforms in the SSA health context be better managed?*

The proposition made in this article is therefore that if a set of guidelines or a framework for developing technology platforms could be developed it may act as a practical and facilitating tool for platform owners to meet the needs of various actors in its ecosystem. In order to develop the tool, the perspectives of different ecosystem actors and the conditions under which they will be able to develop solutions in a platform environment need to be better understood.

The project included a theoretical component with a literature review, leading to preliminary theoretical framework or inventory framework of concepts that could be considered. The subsequent evaluation of the framework entailed a three-step progressive evaluation process namely 1) a theoretical exploratory case study, 2) interviews with industry experts and 3) an application of the framework on an industry-based case study. The following section reflects on the technology platform concept within the ecosystem perspective after which the methodology is discussed in section 3, the evaluation process is discussed in section 4, followed by the proposed framework in section 5.

### The nature of technology platforms

Tiwana, Konsynski and Bush state that, “*the notion of platforms refers to disparate things in marketing (product lines), software engineering (software families), economics (products and services that bring together groups of users in two-sided networks, information systems and industrial organisation*” [16]. Research on platforms often take on one of two perspectives [17, 18]. The first refers to the engineering or technological perspective on platforms [19, 20]. This perspective considers software-based platforms that provide the core architecture on which other modules and extensions can be developed through the use of platform interfaces [16]. In this case the platform is relatively stable and the innovation occurs through the complementary products or services developed using the platform. The second perspective on platforms refers to the economic or market-related view [17, 21] where platforms are viewed as facilitators of interactions between two or more categories of end-users in order to create value [21].

In addition to the two platform perspectives (engineering/economic), four different platform types have also been identified, namely transaction platforms, innovation platforms, integrated platforms and investment platforms^1^ [22]. A transaction platform facilitates transactions through the platform to one or more groups and therefore correlates to the economic or market perspective. Innovation platforms link with the engineering perspective and refer to a foundation on which innovative products, services or technologies are developed. Integrated platforms refer to platforms that are combinations of both transactional and integrated platforms. In their survey, Evans and Gawer [22] found that both innovation and transaction platforms are shifting towards becoming integrated platforms – in essence combining the two perspectives.

This research interprets a technology platform as a combination of innovation and transaction platforms and aims to draw from the synergism between the engineering and economic perspectives. Mindful that a platform can be embedded within other platforms, the platform framework proposed here presents a holistic understanding of platforms by integrating both perspectives in the analytical approach as well as acknowledging specific SSA health-related components into the framework.

To better understand the context of platforms in SSA healthcare, an ecosystems perspective was adopted, a construct that draws from natural ecosystems. This metaphor allows for a useful understanding of ecosystem behaviour and dynamics. It is referred to in management research [23, 24] and provides insights into the relationships between ecosystem actors [25], the ecosystem health [24] and evolution [26]. Thomas and Autio [23] (page 2) define an ecosystem as “*a network of interconnected organisations, organised around a focal firm or platform, which incorporates both production and use side participants”.* A technology platform is therefore a central part of such an ecosystem and vice versa.

In the software industry, firms that are connected by a technological platform form a part of the software ecosystem (SECO). SECOs can be regarded as a subset of business ecosystems [27] and usually increase in value as more users participate on the platform [27, 28]. One of the important differences between SECOs and business ecosystems is that in SECOs, both the ecosystem actors and the software components influence ecosystem health [27]. This research regards a platform ecosystem as the case where the software underpinning the SECO is a technological platform. Likewise, Gawer and Cusumano [29] consider the platform and all interacting stakeholders as the platform ecosystem. Acknowledging Jansen, Finkelstein et al. [30], this research identifies three ‘levels’ of actors in the platform ecosystem, namely the platform owner, the developers and the end users. Platform ecosystems are considered to include all three actors and their relationships, as well as the technology platform and its software components.

The governance of a platform ecosystem is central to its success and is part of the challenges platform owners face. It was therefore a design consideration for the framework developed in this research.

Within the healthcare context, the uptake of platforms are enhanced by various enabling factors (enablers), but simultaneously face barriers to uptake. In general these enabling factors include, convenient to use data, expansion of digital networks, increased connectivity and advances in the Internet of Things (IoT) all contribute to the adoption of such platforms [19, 31]. In an SSA context, the widespread adoption and diffusion of mobile phones as well as advances in Information and communication Technology (ICT) infrastructure are examples of important enablers [32–35]. There are however also significant ICT-related challenges, which include high failure costs [21], levels of regulatory control, data sensitivity, interoperability challenges [36, 37] and data governance [38]. These barriers are underpinned by a number of challenges related to platform implementation in the SSA health context, for example:

- Resourcing challenges including staff shortages and resource constraints, the absence of managers and supervision, insufficient information technology (IT) support, limited funds and infrastructure, power blackouts, digital illiteracy as well as financial, resource and usage sustainability [1, 7, 36, 39–41].
- IT infrastructure challenges comprising limited availability of internet connectivity in some areas, network stability concerns, the inconsistency of infrastructure across locations and infrastructure reliability [1, 7, 36, 39, 42].
- Existing or planned support structures such as the proposed National Health Insurance (NHI) to be implemented in SA, where interoperability challenges and compliance to industry standards are important [36, 39, 40, 42, 43].
- Data collection challenges including the lack of standardisation and interoperability incentives and the need for data quality control [38, 39, 42, 43].

Further work can be done to overcome these challenges in order for technology platforms in the SSA country context could reach its full potential. To tailor the proposed framework for use in the SSA health context, an understanding of the relevant ecosystem and environment is therefore essential.

## Methods

The objective of the research was to develop a framework for the design, development and implementation of technology platforms in the SSA health environment, mindful that the use of technology platforms to address health challenges in this context has not been researched extensively [22].

A Grounded Theory-based approach was followed to develop the platform framework, using the Conceptual Framework Analysis (CFA) proposed by Jabareen [44] as a process comprising of eight steps. Data sources were mapped and concepts identified, deconstructed and categorised. The concepts were then integrated and synthesised into the framework. The CFA process concluded with the evaluation and rethinking of the framework. The eight CFA phases are divided into three main parts, as described in the following sections and summarised in Table 1.

**Table 1:** Overview of the CFA process and its implementation-

CFA eight phases [44]	Objective of phase as per CFA	Alignment with section in this paper	Objectives of part
Phase 1: Mapping data sources	Map spectrum of multidisciplinary literature	Part 1: Investigation and discovery of sources. Subsequent concept identification and categorisation (Process described in Section 3)	- Systematized literature review [45] - Conceptual literature review based on findings of systematized literature review - Include investigation of existing relevant frameworks, models and tools
Phase 2: Reading and categorising of data	Read selected data and categorise by discipline and scale of importance.		
Phase 3: Identifying and naming concepts	Read and re-read data to discover concepts. Allow for concepts to emerge from literature		
Phase 4: Deconstructing and categorising concepts	Identify the main attributes, characteristics, assumptions and role of each concept. This is followed by categorising the concepts accordingly.		
Phase 5: Integrating concepts	Integrate and group together similar concepts to form one group of concepts	Part 2: Development of framework (Process described in Section 3)	- Integrating and synthesising findings into a framework [4]
Phase 6: Synthesis and resynthesis	Synthesise concepts into a framework. This is an iterative process and includes repetitive synthesis and resynthesis.		
Phase 7: Framework evaluation	Establish whether the framework makes sense.	Part 3: Framework evolution (Process described in Section 4) The outcome of the framework evolution process is presented in Section 5	Evaluation and modification of the framework in three stages: - Theoretical case study - Semi-structured interviews - Industry case study This is a continuous process of evolving the framework with the third evolved version framework which is presented in section 5
Phase 8: Rethinking framework	A multidisciplinary framework will always be dynamic and needs to be revised.		

With reference to Part 1 as indicated in Table 1, a systematized literature review was undertaken by the authors to identify previous studies relating to relevant technology platforms, innovation and ecosystems [46], as well as the relevant key concepts. Search terms for publications can be seen in Table 2.

**Table 2:** Systematic literature review search results

Name of Database	Scopus	
Search strategy	Platform AND Technology AND Innovation AND Ecosystem	173
Date of search	30 May 2017	
Years covered by search	No limitation on publication year	

The next phase included choosing the final data sources to be used in the systematized review by assessing the search results against the inclusion criteria^2^ where after it was read and reread in order to characterise the data. The whole process was mainly completed by a single reviewer, with inputs from two super visors who guide the process and continuously reflected on the coding process with the reviewer [44]. The data sources were limited to exclusively English, leaving 173 search results. Thereafter the remaining studies were exported from Scopus into MS Excel for further screening and eventual categorisation. The exported data included: (1) author(s) names, (2) paper title, (3) year of publication, (4) source title (publication/journal), (5) Affiliations, (6) abstract, (7) author keywords and (8) document type.

**Fig. 1:**
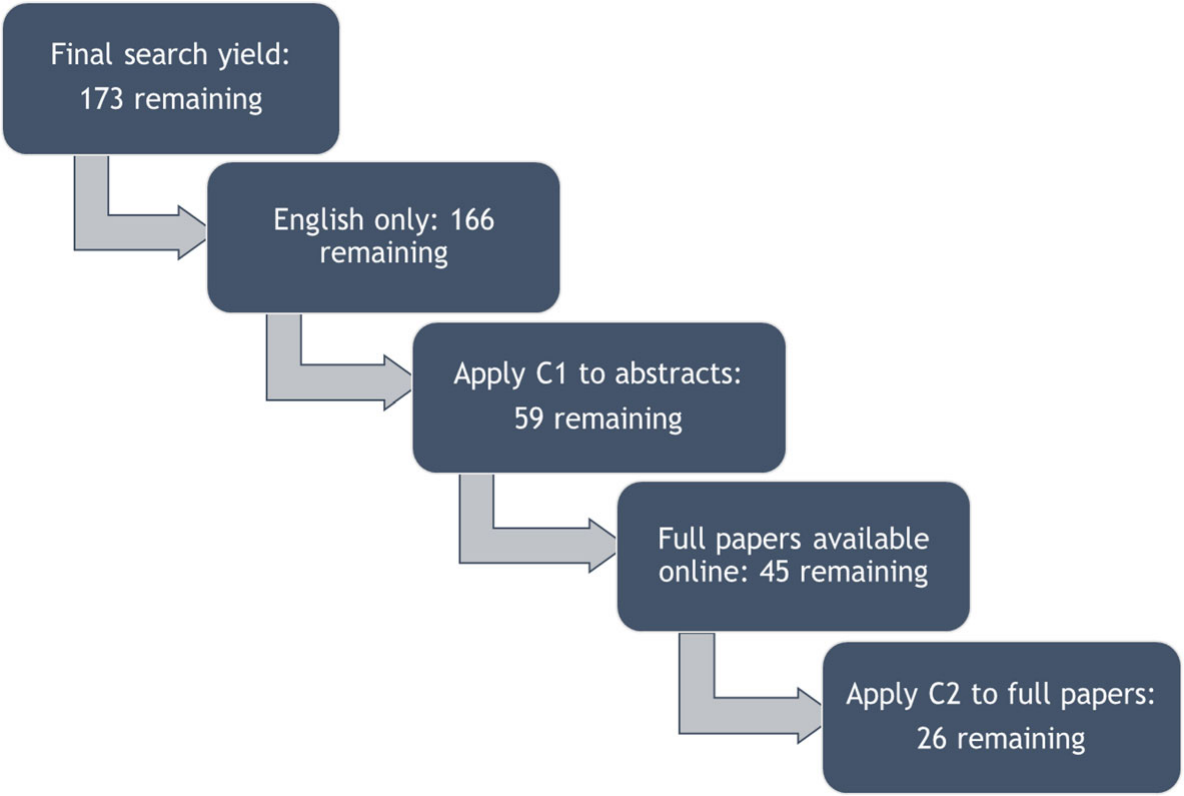
Process of identifying primary studies in systematized literature review. Legend : The systematized process for selecting primary papers for the systematized review. Abstracts were screened according to criteria C1 (removing irrelevant studies based on abstract) and C2 (removing irrelevant studies based on full reading)

The process of identifying the primary papers is illustrated in Fig. 1. As suggested by Petticrew and Roberts [47], the abstracts of the papers were screened and their relevance to the study determined where after the full papers were read and assessed against the original criteria. After applying the category 1 (C1) criteria to the abstracts of the studies and eliminating evident non-relevant studies, all conference reviews and panel discussions amongst other criteria, a total of 59 papers remained. The online availability of these papers was checked and only 45 could be obtained in full text. Books were also excluded as full versions could not be found. Next, the full papers were screened and assessed against the first category (C1) and the second category of criteria (C2). This resulted in the final number of 26 papers which were included. After the initial screening of the abstracts and paper content, the data sources were thoroughly read to allow for an overview of data categories.

The 26 articles identified were therefore analysed and data was extracted by following a 4-step iterative coding procedure developed by [48]. The iterative process involved 1) Primary Cycle Coding to identify high level concepts, 2) Focusing and displaying activities around the first phase coding, 3) Secondary Cycle Coding to develop more detailed categories of higher-level categories and 4) Synthesizing activities. Qualitative analysis software Atlas.ti was used to conduct these various phases of the coding process. This coding process lead to the identification of concepts and patterns in the literature. Through the detailed reading of the final database, each article was critically appraised with respect to its methodological soundness. This aided with any biases and to help the author interpret the data as suggested by Petticrew and Roberts [47]. This phase also included the synthesis of the data by systematically describing, reporting, tabulating, and integrating the results of the studies, which resulted in the deconstruction of each identified concept.

Insights obtained from the review directed the subsequent conceptual literature review viz. (1) key concepts related to this research, (2) the importance ranking of these concepts, (3) challenges facing platform owners, (4) the multi-disciplinary nature of the research, (5) the void of relevant research specifically for the African context, (6) different types of ecosystems and (7) typical ecosystem actors in a platform ecosystem.

The systematized literature review also highlighted the importance of three ecosystem actors, particularly the platform owner, the developers and the end-users:

- The platform owner is the firm responsible for the development of the software, maintaining the software and governing the ecosystem.
- The developers refer to the complementors or innovators who develop complementary products, services or technologies using the technology platform.
- The end-users are the final users of the applications, extensions or modules developed by the developers using the platform.

The ecosystem actors, their roles and how the key concepts relate to each of the ecosystem actors are shown in Table 2. A clear understanding of each actor and focus areas were key for the ecosystem viewpoint of framework development.

Subsequently, Part 2 of the process entailed the framework that was formulated (relating to phases five and six of the CFA process (as discussed in Table 1). The framework was developed through an evolutionary process, commencing with a preliminary framework which was evaluated and modified in three stages as shown in Table 3. The preliminary framework comprised an inventory list of concepts for each ecosystem level (shown in Appendix A) and formed the foundation of the framework itself.

**Table 3:** Summary of the review - Descriptions and key focus areas of ecosystem actors

Level	Description of level	Level specific focus criteria (from literature reviews)	Key references
Platform owner	The owner and designer of the platform. The firm manages the platform and its boundary resources. Typically responsible for the governance of the ecosystem.	Platform design, platform and ecosystem management, value creation, platform architecture, evolution, competition, openness, control, entry barriers, governance	[16, 18, 49–53]
Developer	The developers of the software products, services or technologies (applications) using the platform. They can be within the platform owner firm (internal platform) or third-party companies (external platform).	Boundary resources and usability, accessibility, entry barriers, ability to innovate	[51, 54–57]
End user	The end users of the products, services or technologies (applications) developed using the platform.	Usability, accessibility, cost	[56, 58, 59]
Ecosystem	The platform ecosystem comprises the platform owner (including the platform), developers and end-users. It is formed around the central platform.	Health, value co-creation, governance, evolution, control, entry barriers	[16, 24, 60–62]

**Fig. 2:**
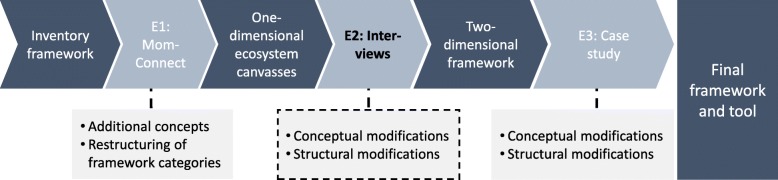
Process of framework evaluation through three stages (E1 to E3). Legend: Figure 2 show the process of framework evaluation that was completed through three stages namely (1) a theoretical case study on an existing health platform in the SSA context (the MomConnect project) (2) local and international semi-structured interviews and (3) an industry case study

The final component of the framework development process focused on an evaluation and is discussed in the following section and summarised in Table 3. The evaluation comprised three stages, viz. (1) a theoretical case study on an existing health platform in the SSA context, (2) local and international semi-structured interviews and (3) an industry case study (See Fig. 2).

## Framework evolution

This section presents the process and outcomes of the three evaluation stages undertaken to develop new evolutions of the framework, due to space limitations we briefly present the main findings from these stages and then proceed to describe the evaluated framework in section 5.

### Stage 1: Theoretical case study on MomConnect

The first stage of the framework evaluation process included an exploratory case study on a platform-based digital health platform initiative (Fully presented in [63]). The case study conducted in this research is classified as a theoretical exploratory case study. A detailed process for conducting case studies as proposed by [64] was followed: 1) Designing the case study protocol, 2) Conducting the case study, 3) Analyse the case study evidence and 4) Develop conclusions. The approach taken for the case study was to theoretically from secondary sources investigate and understand how the framework components can be recognised in a real-world platform level initiative. The aims of this case study was thus to form the first stage of framework evaluation and to gain a better understanding of how the framework may help to understand the functioning of a technology platform in the SSA health context. This enabled a preliminary validation of the existing concepts within the inventory framework (see Appendix A). This also provided the opportunity to include additional concepts found in the case study into the inventory framework and therefore enabled the modification and adaptation of the original concepts.

The investigation was conducted on a National Department of Health (NDoH) initiative - the MomConnect digital health platform. The MomConnect programme is a comprehensive digital health program, launched in SA in 2014. The objectives of the MomConnect platform are *“[to deliver] targeted stage-based health information to pregnant and postpartum women, [to] enable women to reach out with pressing questions, and [to] establish an important feedback loop to improve services* [33]*”*.

MomConnect case study was selected based on three main reasons: 1) The success and scale of the platform – it is one of very few platform initiatives at the time to achieve national scale, registering 500,000 women in its first year of operation which was more than 50% of the pregnant women served by the SA public sector; 2) The platform operational context was South Africa and 3) There was good data availability of the platform initiative to enable a case study based on secondary sources [65, 66].

The case was thematically investigated with regard to its strategic management, the technology platform and architecture as well as its user-centric design approach. The findings were related to the three ecosystem levels of the framework, i.e. platform owners, developers and end-users. The various elements of the framework were tested against the case study to see where thy are present and where there were additional components that had to be added. From this preliminary evaluation it became evident that the inventory framework could be successfully applied to a developing country health platform. This provided a starting point for tailoring the inventory framework specifically to the SA health context.

The application proved that the inventory framework could provide useful insight into the concepts a platform owner should consider in the design and management of a platform and its ecosystem. At the platform owner level, MomConnect could be linked to the strategy, architecture, governance structure, internal organisation and operations categories. Approximately all concepts within the first two categories could be confirmed to be present in the MomConnect platform. The developer level of the framework also applies to the platform owner. Factors regarding the entry barriers, ecosystem-related concepts, the architecture, support structures and methods of control of the MomConnect initiative were identified. As the platform involves multiple partners and stakeholders, there were concepts that related to an ecosystem perspective. The initiative also leveraged the innovative skills of public and academic partners and allowed them to share innovations. The third and final level of the inventory framework related to end users refers to the mothers, caretakers and the healthcare workers. The categories that could be applied to the MomConnect platform included the context of use, ensuring quality during app use, enabling user feedback, and considering the attractiveness of the app for end users.

The theoretical insight obtained during the preliminary evaluation of the inventory framework, it also led to the reconstruction of the categorisation within each of the three levels (See Appendix B). The platform owner level was adapted by transforming the five overarching categories of the inventory framework into four categories and corresponding subcategories. The resulting main categories were platform design, platform ecosystem design, platform owner design and evolution. These categories were found to be more descriptive and comprehensive. The developer level of the framework was transformed from having seven categories into five categories which were entry barriers, ecosystem, technology infrastructure, control and support. The final level of the inventory framework, the end-user level, experienced the least changes. The five overarching categories were adapted to form three categories with subcategories where applicable. The main categories were context of use, quality control and interface and design.

### Stage 2: Semi-structured interviews

Semi-structured interviews were conducted to further validate the concepts of the framework. The nine participants included platform owners and developers as well as experts in health, ecosystem governance and technological innovation. The reason for the variety of interviewees was to obtain vast perspectives in this initial part of the validation process. Although this is admittedly a small sample size, the process was not a statistical one, but a process to gain insight into the core components of platform management and the applicability and validity of the framework in the platform and platform ecosystem context. The principle of data saturation was followed which settled on this number of interviews.

**Fig. 3:**
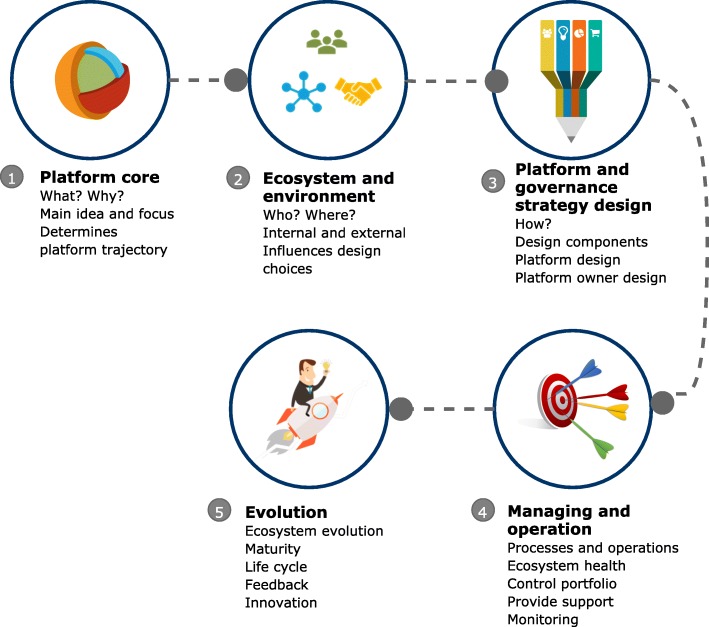
Five overarching parts to the interviews. Legend: Figure 3 show five overarching part of the interview process. Respondents were asked to comment on issues relevant to (1) platform core, (2) ecosystem and environment, (3) platform design and governance, (4) managing and operation and (5) how the process of evolution was managed

Rabionet’s [67] interview protocol was followed where (1) interviewer introduction and background was provide through a short MS PowerPoint presentation, and (2) the interview questions were posed. With more than one hundred concepts distributed throughout the three different ecosystem levels of the framework. Asking one hundred questions of an interviewee is not practical. The researcher formulated a discussion guideline to cover five platform development parts: (1) platform core, (2) ecosystem and environment, (3) platform design and governance, (4) managing and operation and (5) evolution (See Fig. 3).

The subsequent data analysis included three coding cycles, each with specific outcomes and conclusions, shown in Table 4.

**Table 4:** Framework evaluation overview and outcomes

Evaluation stage	Framework evolution	Overview of evaluation stage	Outcomes of evaluation stage
Stage 1: Theoretical case study on MomConnect	Inventory framework	Research an existing health platform in the SSA context and relate it to the inventory framework.	- Verify the applicability of current framework content - Gain insight into technology platform operation and the SSA health context
Stage 2: Semi-structured interviews	One-dimensional ecosystem framework	Semi—structured interviews with industry experts in both the local and international contexts.	- Verify all concepts within the framework - Understand design, development and ecosystem governance - Obtain feedback from industry experts
Stage 3: Industry case study	Two-dimensional framework	Investigate and conduct interviews with a platform firm operating the SSA health context to evaluate the content and usefulness of the framework.	- Observe and investigate the functioning of a platform in SSA health context - Evaluate the usefulness of the framework as a tool - Obtain feedback on tool

The first cycle coding was conducted on paper and in MS Excel. The approach was to go through each interview’s data and to mark which of the concepts were validated. This was done for all the interviews independently. The interview data in MS Excel was formulated in such a way as to enable the recording of the amount of times each concept in the framework was mentioned or discussed by interviewees. By tabulating the concepts and sorting them in descending order, trends in popular concepts could be identified and interpreted.

The second cycle of coding adopted five lenses derived from the notes and highlights of the first coding cycle. The aim of this cycle is for further refinement and investigation of any additional concepts that should be added to the framework. Five lenses were adopted for the second cycle coding: (1) health-related, (2) SSA considerations, (3) platform control, (4) support structures and (5) financing and pricing related aspects. The second cycle of coding also pursued the identification of voids in the framework, highlighting of disagreements and the identification of additional concepts to add to the framework. The five platform development parts and the five lenses formed the basis of identifying the voids and additional concepts to include in the framework. The qualitative analysis process considered the interviews against the framework for (1) confirmed topics, (2) voids and disagreements, (3) additional concepts and then (4) relating to the five lenses, if applicable.

The third and final cycle of coding yielded themes, patterns and deeper insights into the data building on the outcomes of the previous two cycles. In the previous two cycles there were certain topics that featured continuously throughout the interviews. These were identified as trends and patterns that should be considered when designing, developing and implementing a technology platform in the SA health context.

### Stage 3: Industry case study

The third and final stage of the framework evaluation process included an in-depth industry case study on a platform-based firm in the SSA context, Mezzanine ware. The case study conducted in this research is classified as an explanatory case study. A detailed process for conducting case studies proposed by [64] was followed: 1) designing the case study protocol, 2) Conducting the case study, 3) Analyse the case study evidence and 4) Develop conclusions, recommendations and implications. The approach taken for the case study interviews was to investigate and understand how Mezzanine Ware operates, how they are managed and subsequently relate this back to the framework. The interviews for this case study were semi-structured and the predetermined questions were derived from the framework. Interviews were conducted in two stages: (1) relating to the ecosystem dimension of the framework and (2) relating to the platform development dimension. The approach was to prompt the interviewee to discuss the overarching categories of each of these dimensions in order to gain an understanding of how Mezzanine Ware operates in each of the categories.

Data sources included the firm’s website, news articles, published material, organisational notes as well as interviews with five employees and the CEO.

As with the previous two evaluation stages, results-based modifications and adaptations were made to the framework. Structural modifications included the renaming and re-categorisation of several categories and concepts throughout the framework, adding a feedback loop to the platform development dimension and a segmentation of the end-user level. The modifications of the framework included the addition of a combination of 26 concepts and tools related to both the ecosystem and platform development dimensions.

The segmentation of the end-user level of the framework into two user-groups was a significant change. In the SSA health context, wide spread poverty and the digital divide means that the actual end-users of applications are often not well informed regarding the possibilities and impact of the technology and often cannot afford the application themselves [68–70]. A client or intermediary then acts as the middle-man between the platform firm and the actual end-users. This client identifies the need in a community and the potential solutions provided by the technology and then promotes and sponsors the initiative. The end-user level of the framework was modified to account for this use-case. Results: Proposed framework for the design, development and implementation of technology platforms in the SSA health context.

The proposed framework for health-related platforms has four overarching aims, namely to 1) act as a practical and facilitating tool for platform owners; 2) account for each of the three ecosystem levels at the design stage; 3) highlight and address a number of challenges relating to each ecosystem level and 4) integrate the economic and engineering platforms perspectives into an overarching management tool.

**Fig. 4:**
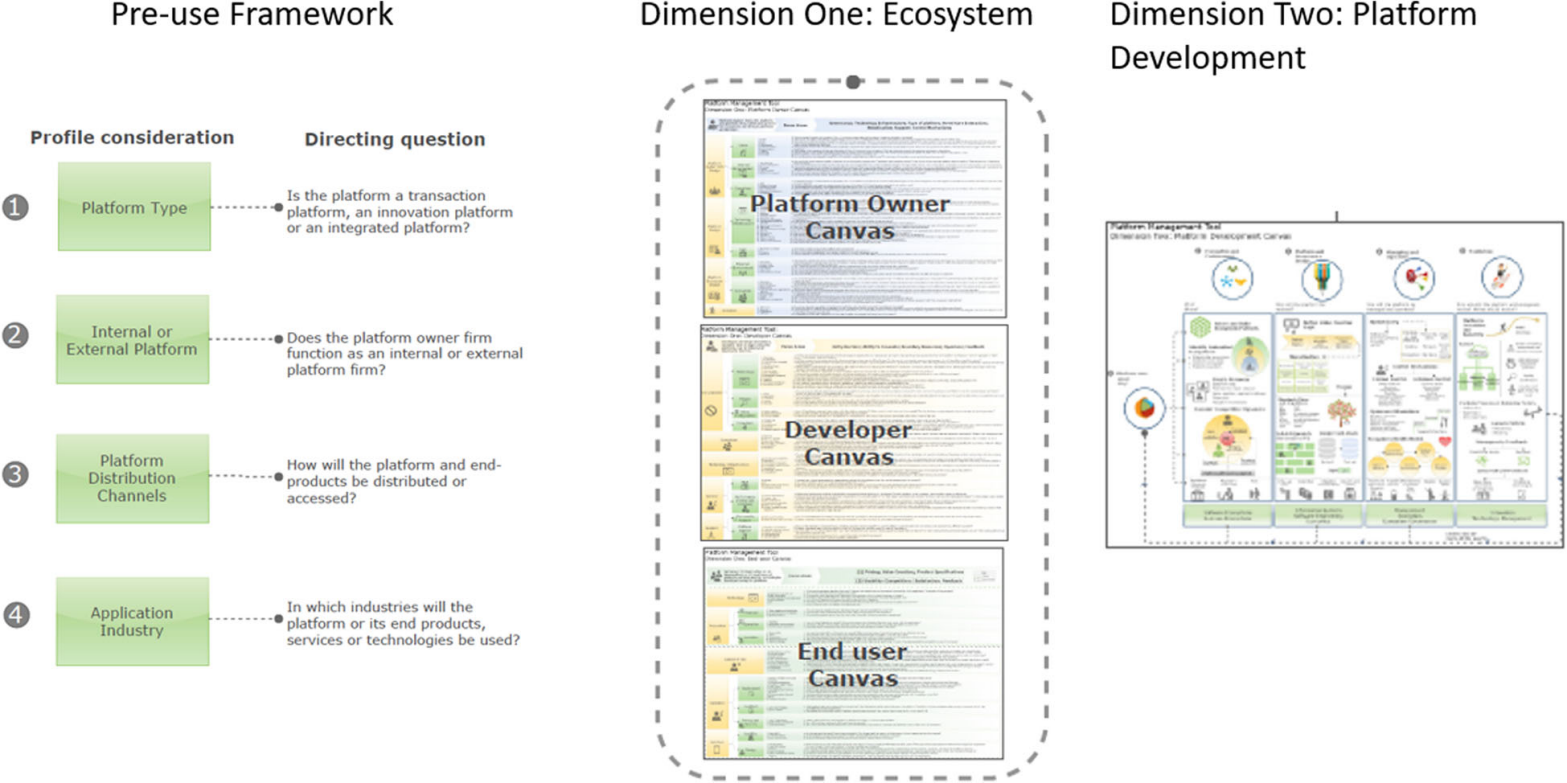
Overview of the dimensions and canvasses in the framework. Legend: Figure 4 shows the framework that was developed which consist of three components namely the 1) pre-use component 2) ecosystem dimension and the 3) platform development dimension

As shown in Fig. 4, the subsequent developed and evaluated framework comprised three components described below, namely the pre-use component, which includes four considerations the platform owner should be aware of prior to using the framework. Then the framework comprises of two dimensions, namely an ecosystem dimension and the platform development dimension.

The purpose of the ecosystem dimension (*Dimension One*) is to guide the platform owner regarding various issues to consider from the ecosystem perspective of the different groups to consider before embarking on designing a platform (See section 4.2). Three perspectives are included namely the platform owner, developer and end-user perspectives.

*Dimension Two* outlines typical platform development components and presents an interpretation of the viewpoints included in the ecosystem levels (Dimension One). This second dimension also brings in specific aspects related to geography (SSA) and application industry (health). Dimension Two comprises of five parts, viz. 1) Establishing the platform core 2) The ecosystem and environment 3) The platform governance and design and 4) Managing and operation of the platform and ecosystem and 5) Evolution of the platform and ecosystem (See section 4.3).

### Pre-use component

During the framework development process, particularly the evaluation process, the importance of defining the platform characteristics became evident. Especially throughout the interview process, the authors realised that clearly defining the platform was an essential step to ensure that the user has the correct perspective when using the framework and understands his own point of reference within the larger platform definition. Four elements that a platform owner should establish regarding the platform under consideration were identified (and indicated in Table 5), viz.

- The platform type should be determined, i.e. transactional, innovation or integrated platform as defined previously.
- It should be established whether the platform firm functions as an internal or external platform. This specifically has an effect on the developer and end-user levels of the framework.
- The desired distribution channels and context(s) of operation should be identified.
- The application industry in which the platform will operate should be specified.

**Table 5:** Final framework: Establishing the platform profile

Platform Profile consideration	Effect on use
Platform type	Platform type results in to one of the following cases: • Elements relating to economic perspective accentuated • Elements relating to engineering perspective accentuated • Elements relating to both perspectives accentuated
Internal or external platform	Internal or external will result in the following two cases: • In an internal platform, platform owner and developer levels merge • In an external platform, the platform owner firm and developers are different firms and the framework levels should be approached accordingly
Distribution channels and contexts	Depending on the desired distribution channels and contexts, certain elements will be emphasised. For example: decisions regarding cloud-based, online or offline access or distribution via a marketplace (Appstore, for example) will affect elements applicable within framework.
Application industry	Certain industries (e.g. health) will require special attention to certain categories and concepts. For example relating to standards, protocols, control mechanisms, context of use etc.

### Dimension one: ecosystem actor levels

**Fig. 5:**
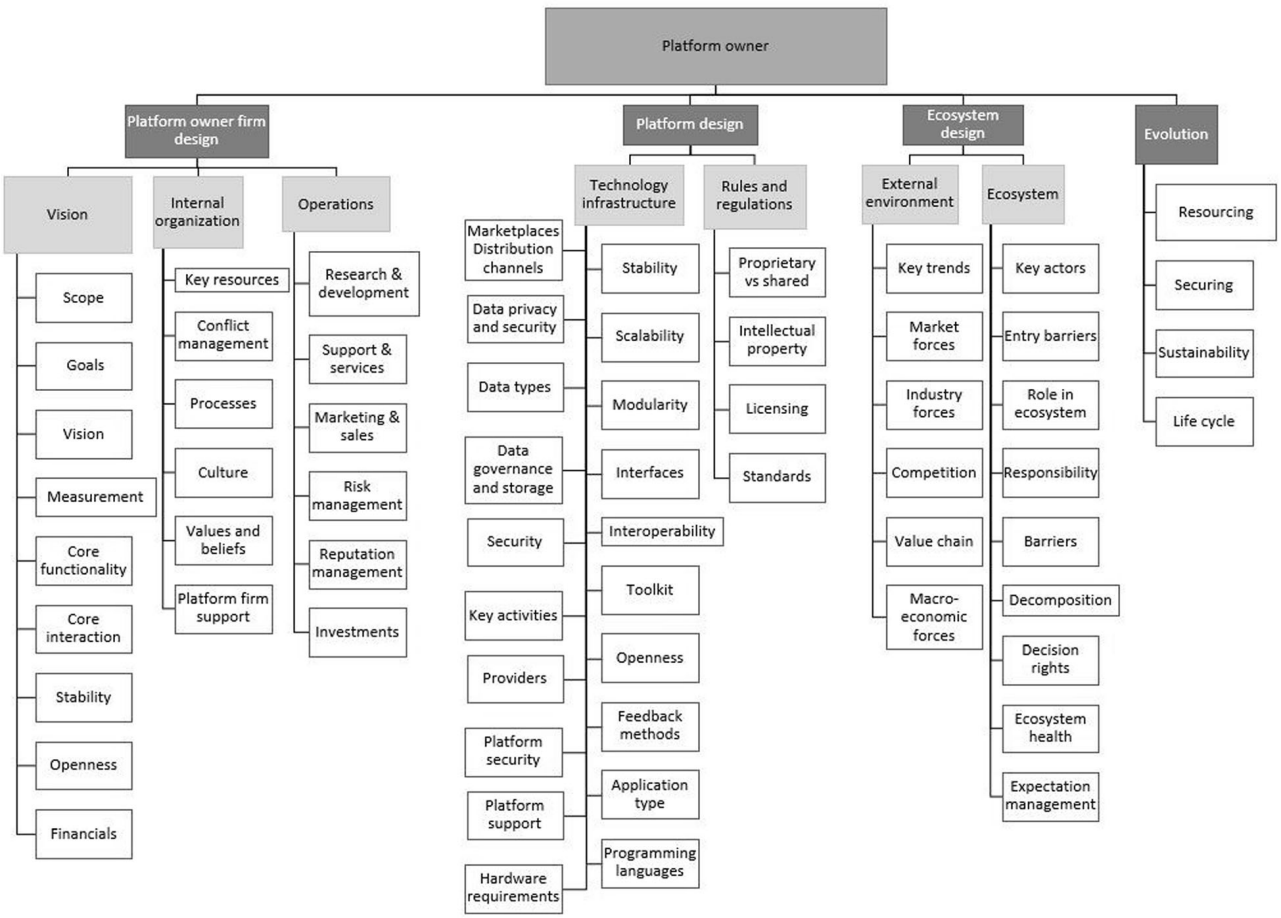
Framework - Dimension one: Platform owner level. Legend: The platform owner level is shown in Fig. 2 and comprises of four main categories, namely 1) Platform owner firm design 2) Platform design 3) Platform ecosystem design 4) Evolution of the platform and ecosystem

**Fig. 6:**
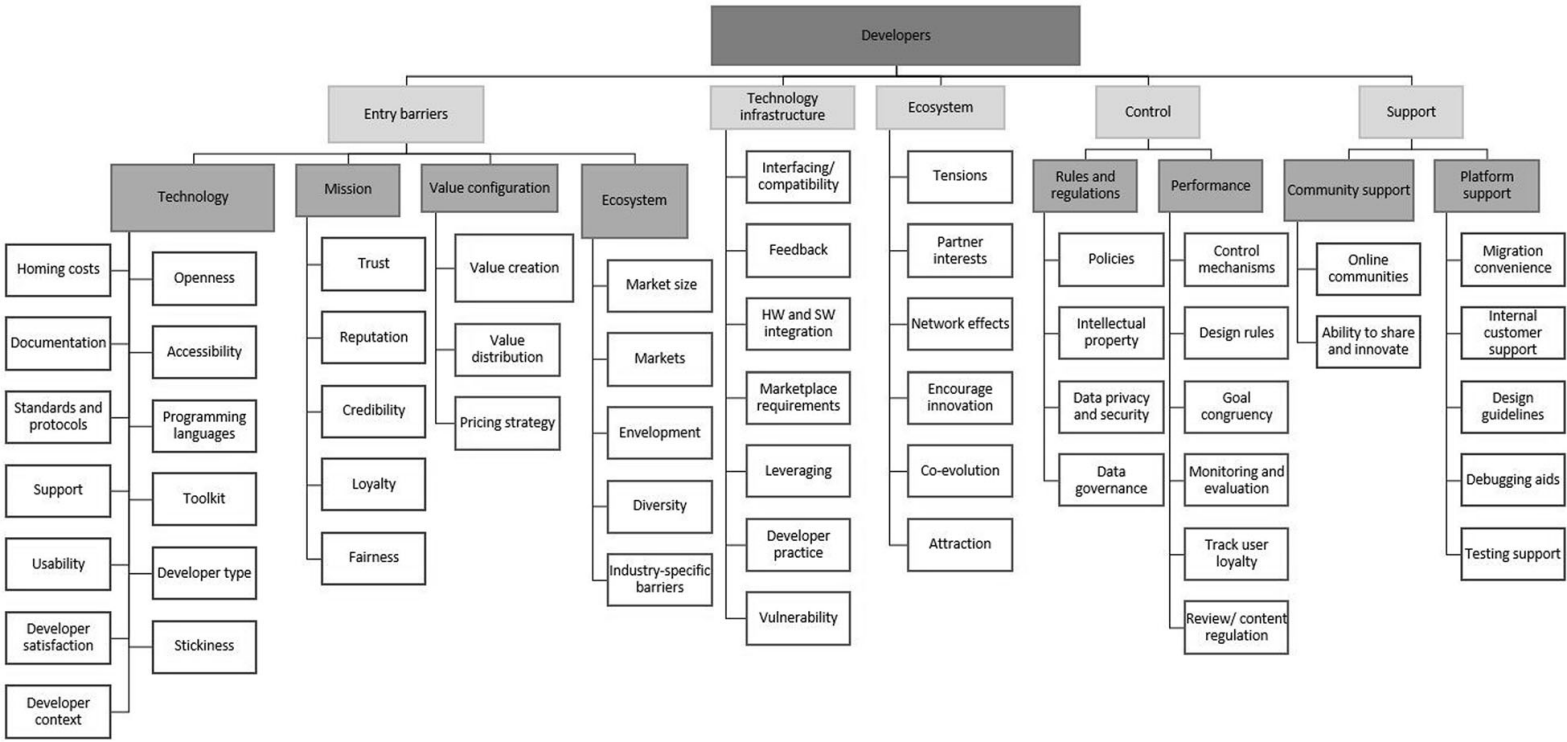
Framework - Dimension one: Developer level. Legend: Figure 6 shows the developer level can be segmented into five areas namely 1) entry barriers 2) technology infrastructure 3) ecosystem 4) control 5) support

The ecosystem dimension of the framework comprises of three levels (indicated in Figs. 4, 5 and 6), viz.

- Platform owner level
- Developer level
- End-user level

These levels highlight key concepts that a platform owner should consider with regard to each of the ecosystem levels. The platform owner can then formulate key questions regarding each concept to guide the process of design, development and implementation of the platform and ecosystem.

#### Platform owner level

In our framework we consider the platform owner as the firm that is responsible for the design of the platform architecture as well as the building and governance of the platform ecosystem [16]. The platform owner must address governance, the technology infrastructure, establishment of the platform profile, monetisation as well as support and control mechanisms. The platform owner level is shown in Fig. 5 and comprises of four main categories, viz.

- Platform owner firm design
- Platform design
- Platform ecosystem design
- Evolution of the platform and ecosystem

‘Platform owner firm design’ is subcategorised into three elements, viz. platform vision, the firm’s internal organisation and its operations. The vision refers to the importance of defining a scope [71, 29], establishing goals and the subsequent measurement strategies [51, 72]. The core interaction [53] facilitated by the platform as well as its core functionalities should be established early on as it will have effects on the design and management of the platform and ecosystem. Sustainable sources of funding are imperative. The platform owner should also decide on the level of openness it plans on adopting [51, 71].

The internal organisation element of the ‘platform owner firm design’ element focuses on the internal workings of the firm. This includes the identification of the key resources required to successfully design and operate the platform and ecosystem, as well as how to manage the potential conflict between both the resources and ecosystem. Company culture, values and beliefs should also be determined as they may have an effect on the wider ecosystem [16, 28, 51]. The final element is the operations of the firm to ensure the successful operation of the platform and wider ecosystem. These include research and development, support and services, marketing and sales [50, 72], risk [73] and reputation management [29, 71, 74] and financial investments [29, 75] into the firm and ecosystem.

The second category with regard to the ‘platform owner level’ is platform design and has two subcategories, viz. technology infrastructure and rules and regulations. The technology infrastructure category refers to concepts which need to be considered in the design of the technology itself. They are mainly based on general design principles and developer entry barriers. The core platform should be stable, whilst allowing modularity at its interfaces [18, 54, 76]. It may also have to be scalable [28] and interoperable with other systems or technologies. The type of application (mobile, web-based or hybrid applications) [77] of the platform and its end-products, services or technologies also has an effect on the design of the platform.

Platform owners should be mindful of issues which the developers deem important, such as platform openness [20, 78], feedback methods [24, 79], programming languages [51] and toolkit elements [51, 55, 80]. Developers need to contrite to the platform as innovators and if the platform satisfy their needs they are more likely to be willing to choose this environment to develop applications.

As indicated in the Pre-use section above, the industry or context may require specific data privacy, security and governance or storage methods. Security includes that of the data but also of the platform itself, especially if the platform is an external platform and its boundaries are open [21]. External platforms are open for use from external innovators which means that the boundaries of contributors may be more open. Potential providers [53] as well as hardware requirements are additional important considerations. Diverse hardware devices would potentially require certain software adjustments relating to screen size, operating system, etc. to ensure smooth operation.

‘Platform ecosystem design’ is the third category and comprises of two subcategories. The first subcategory refers to the environment external to the platform and ecosystem over which the platform owner has no control. Elements to consider include technological and consumer trends, market and industry forces, competition, possible value chain influences and macro-economic forces [81]. This may for instance refer to the external environment within a supply chain visibility platform initiative where these issues are outside the control of the platform owner but still impacts on the ability to implement the innovation. The second subcategory refers to the platform ecosystem including the key actors within the ecosystem, defining their roles, responsibilities [16], expectations, entry barriers [72] [51, 74] and decision rights. Here again referring to the supply chain visibility platform technology various supply chain participants in the supply network have different capacities, resource levels and roles to fulfil, ranging from global manufacturers of vaccines, to clinic managers in rural areas. The platform owner may decide to decompose the ecosystem into subsystems for easier management [16]. This may mean that the platform is divided into sub platforms for various supply network actors and provide specific services to them as needed. The platform owner is also responsible for the ecosystem health and should investigate methods of evaluation and measuring the ecosystem health.

The final category on the platform owner level refers to the ‘evolution of the platform and ecosystem’. This includes the continuous addition of new resources and subsequently securing the platform [55]. Healthcare platforms may utilise sensitive data for which appropriate security measures will be absolutely crucial. It also highlights the need to design for sustainability in terms of the technology, the ecosystem and the platform firm itself. The final element in this category refers to the life-cycle of the platform and how this may influence the managerial focus of the platform owner at each of the different life-cycle stages.

#### Developer level

The developers are the actors who develop the technologies, services or complementary products using the platform [19]. They can be external or internal to the platform owner firm. A platform owner may consider entry barriers, innovation, boundary resources, platform openness and ability to give feedback as focus areas concerning the developers using the platform. The developer level can be segmented into five categories as shown in Fig. 5.

The first category on the developer level refers to ‘entry barriers’ in terms of the technology involved, the nature of the firm itself and how it is perceived, the value configuration and the platform ecosystem. Developers are the main sources of value creation with regard to platforms and therefore there are entry barriers regarding the value configuration. These include the value creation and distribution within the ecosystem as well as the pricing strategy the platform owner decides to implement [79, 82].

The relevant entry barriers are considered based on typical concerns and considerations developers have when joining or leaving a platform and ecosystem. The level of openness of the platform and platform firm influences the motivation to join, as it has a direct effect on the level of innovation that a developer can attain [74]. It is recommended that the platform be accessible, use popular programming languages [51, 80] and standard protocols, and that support is provided in terms of a toolkit and documentation. The platform owner should consider the user-friendliness of the platform and what level of satisfaction it will provide developers. Developers are often hesitant towards lock-in and therefore stickiness [27] and homing costs [16] should be accounted for. Specifically in SSA, the context of the developers should be considered. For example, the latest technologies or resources may not be available or connectivity may be limited.

Developers may also be influenced by how the platform firm is perceived and therefore the second subcategory of entry barriers refers to the firm’s mission. The platform firm should work towards fostering a sense of trust, cultivating a good reputation [52] and credibility and focus on being loyal and fair to all actors within the ecosystem [80].

Aspects of wider ecosystem can also become entry barriers. Developers may look towards the wider market size [51, 75, 80] that they will be able to reach through the platform, the different marketplaces available [75], the possibility of envelopment [19] and the diversity within the ecosystem [62]. There may also be specific industry-specific elements that give rise to resistance to adoption. In healthcare industry, for example, there are high levels of security and privacy which need to be respected and adhered to.

For the second category, the developers are also considered in terms of the technology infrastructure of the platform. In order to co-evolve with the developers, encourage innovation and reduce possible tensions, the developers should have mechanisms to influence what should change on the platform. This also relates back to designing the platform to focus on usability not only for end users, but also for the developers. Feedback [50] therefore forms a crucial part of a developer’s role and their opinions are reflected in the platform software’s version updates. Different hardware components might require specific adaptations in software (for example, screen resolution of different mobile phones) [51]. There are also mechanisms to support good developer practice and to reduce vulnerabilities such as possible weak points in the software especially when working with sensitive data such as healthcare related data that may include patient records [83].

The third category on the developer level refers to ‘ecosystem considerations’, specifically those relating to the developers within the ecosystem. There could potentially be tensions between the developers or between a developer and the platform firm, which the platform owner needs to address [16, 28], perhaps arising due to envelopment of developer functionality to insufficient diversity between developers. The ecosystem should also be designed, managed and governed to account for the interests of all the stakeholders involved [73], encouraging innovation [74, 79] and network effects. The platform owner should be mindful about methods of attracting developers and to facilitate the co-evolution between developers and the platform [62].

The final two categories of the developer level of the framework (‘control’ and ‘support’) were addressed during the evaluation phase of the framework.

The category of ‘control’ has two subcategories, viz. rules and regulations and performance. Rules and regulations refer to concepts such as policies applicable to platform use or developer end-products, intellectual property rights of both the platform and developer complementary products, services or technologies [28, 29]. It also includes the privacy, security and governance decisions regarding the developers, their innovations and the data involved [21]. Performance-related concepts include establishing and enforcing control mechanisms [16, 79] and design rules [16], determining to what extent goal congruency is required throughout the ecosystem [79]. It also refers to the monitoring and evaluation of the developer performance, the need for reviewing the products services or technologies or enforcing content regulations [58, 80]. A platform owner may also consider tracking developer loyalty [75]. If a platform owner notices a specific group of developers are leaving the platform simultaneously, for example, it may indicate the rise of a competing platform ecosystem.

The final category developer-level category is ‘support’ and comprises community and platform support sub-categories. Support for all ecosystem actors has shown to be key in platform and ecosystem success. Firstly, a platform owner can motivate or facilitate external communities providing platform support [72]. The platform and platform firm should also provide a significant amount of support to developers. Considerations include easing the migration convenience from other platforms, having a dedicated internal customer support team, providing support in the design guidelines and providing or recommending debugging and testing support [77, 80].

#### End-user level

The third level of the framework refers to the platform ecosystem end-users and is shown in Fig. 3. It is divided into two groups, viz.

- Client group: Dedicated to the clients who typically act as intermediaries between the platform firm and a group of end-users. Pricing, value creation and the precise product or service specifications are some of the focus areas of the client.
- Actual end-users’ group: End-users of the products, services or technologies developed using the platform and usability, competition where user satisfaction and feedback are important.

To illustrate the roles of the two groups, consider for example the case where the government of a SSA country identifies the need for a digital health tool in a government hospital. The hospital itself does not necessarily know of the benefits of such a tool and cannot pay for it, therefore the government acts as an intermediary.

The end-user level may not always be completely applicable to platform owners. If the platform is an external platform firm, the platform owner may not have any effect on the end-users [29]. However, in the case of an internal platform, the end-products, services or technologies are developer within the platform owner’s firm and therefore the end-user level becomes significant.

##### Client group

Two main categories, viz. technology and the proposition-related concepts, are relevant to the client group of the end-user level. ‘Technology’-related concepts include the desired application type and its envisaged use and establishing how the data gathered will be used and governed. The rules, regulations and standards related to the use-case should also be noted [24]. The platform owner should consider any existing systems or databases that the end-product, service or technology might have to interoperate with. In SSA countries, existing databases are often siloed and operating systems might be outdated [6, 84].

The ‘proposition’ category relevant to the client group has three sub-categories which should all be understood prior to designing the end product, service or technology: financial, operational and evolution. Financial concepts refer to how economic value will be created and distributed between the client, actual end-users, developers and platform [55]. The initiative may require significant initial investments and risk and these should be accounted for in the monetisation strategy. There should also be clarity on the expected returns of the initiative. Operational concepts include active feedback methods between the client and platform, as well as implementing the desired feedback between the end-users and the clients [50]. This latter feedback may be required for the improvement of specific services such as healthcare in clinics or hospitals [35]. During the evaluation of the framework it was highlighted that communication channels may have to be facilitated through the platform and should therefore be incorporated in the design. The client may also desire certain monitoring and evaluation mechanisms incorporated into the software.

Finally, with regard to the ‘proposition’ category, there should be clarity regarding the evolution of the product, service or technology. Sustainability is one of the most important concepts, especially within the SSA context. The platform should be sustainable in terms of the technology itself, the financial considerations, the client and platform owner’s firms as well as sustained use [6].

The evaluation of the framework suggested that the platform owner should consider the relevance and significance of the client in terms of its long-term vision. A ‘wrong’ client may result in scope creep or misaligning the firm with its vision and goals. However, the opposite may also be true. In the SSA health context, partnering with large and influential organisations may open numerous doors in future. Acknowledging that technology is constantly evolving, it follows that the end-product and service will also need to evolve. Hence, there should be clarity on the co-evolution between the client, the platform firm, the applications and the platform and whether the software may be re-used for other projects with different clients.

##### End-user group

The second group of end-users refer to the actual end-users of the products, services or technologies. This group is divided into three main categories, viz.

according to the context of use, the operation and the interface considerations.

Context of use includes organisational, physical, social and geographic contexts which may all have an effect on the design of the application [85, 86]. Understanding the task and user characteristics are also vital prior to the design [86]. The platform owner should consider how the users in their envisaged contexts will access the application and consider whether there will be different managerial or hierarchical levels of these end-users, as done with the MomConnect platform [87]. This refers to the case where the application is for example deployed in a hospital, but the nurses, doctors and hospital managers will use the application and therefore might lead to different design aspects.

**Fig. 7:**
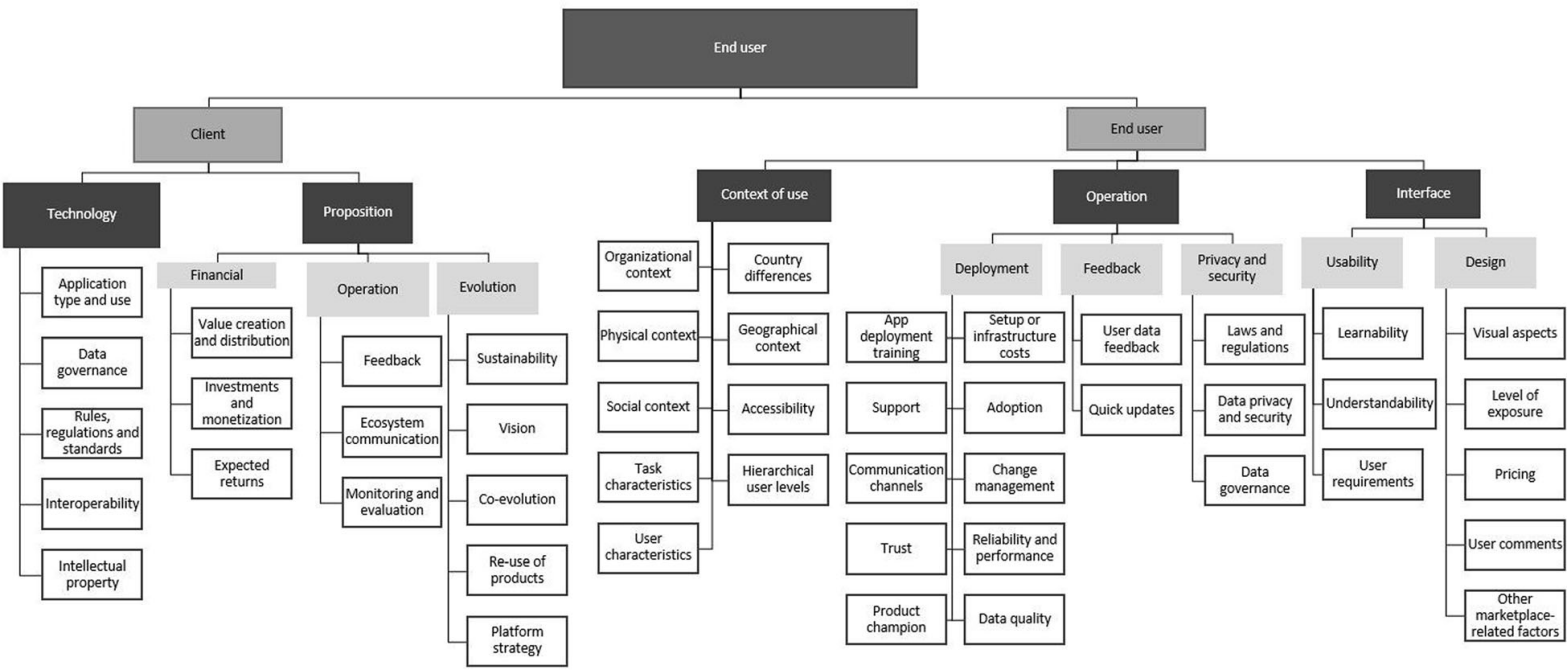
Framework - Dimension one: End-user level. Legend: Figure 7 show the platform ecosystem end-users which is divided into two groups 1) Client group who typically act as intermediaries between the platform firm and a group of end-users. 2) End-users of the products, services or technologies developed using the platform and usability, competition where user satisfaction and feedback are important

The operation category comprises deployment, feedback and privacy and security-related concepts. Deployment refers to the releasing of the application to the end-users and includes the infrastructure or setup costs, ensuring adoption, facilitating change management and providing deployment training and support if required [84]. All these concepts are all particularly significant in the SSA health context. Sufficient communication channels should be enabled and a sense of trust might be required for comprehensive and sustained end-user adoption. The identification of a product champion may be beneficial for both communication with the end-user group and fostering a sense of trust. The application designer should ensure the quality of data gathered is sufficient [3] and that it is reliable and performs desirably [85, 88] (Fig. 7).

Feedback and privacy and security are vital, specifically in the SSA health context. User data should be collected and used to implement rapid updates if required. The data of all end-users should be protected and subjected to stringent data governance, as it will typically be sensitive and personal health data [58, 89].

The final category of the end-user level refers to the interface of the application. The interface is divided into the usability and design considerations. The application developer should ensure the learnability and understandability of the application whilst meeting all user requirements [85]. The interface design should also be visually pleasing [85, 90] and enable user comments [90, 91]. The pricing of the application can also be fundamental in its success and should be aligned with the platform strategy [90].

This concludes the ecosystem dimension of the framework and the second dimension focuses on platform development.

### Dimension two: platform development

The second dimension outlines typical platform development components and presents an interpretation of the concepts included in the ecosystem levels (Dimension One). This second dimension also incorporates specific aspects related to SSA and health. The ‘platform development’ dimension comprises of five parts, viz.

- Establishing the platform core
- The ecosystem and environment
- The platform governance and design
- Managing and operation of the platform and ecosystem
- Evolution of the platform and ecosystem.

The aim of Dimension Two^3^ is to structure the thinking that underpin the platform development and to provide tools, methods or approaches that a platform owner could use in the different development parts. Each of the concepts in the levels indicated in Dimension One can be mapped to one or more of the five platform development parts.

The ‘platform core’ part refers to the ‘what’ and ‘why’ considerations and does not include any sub-elements and forms the core of the platform development.

The ‘ecosystem and environment’ part refers to the ‘who’ and ‘where’ of platform development. It includes four general elements and three SSA specific health-related elements. Platform owners should carefully select their ecosystem partners and actively build the ecosystem based on this vision. Depending on the platform profile, the platform may form a part of more than one ecosystem. This can lead to embedded ecosystems, such as the ecosystem involved with a specific project, which is embedded within the larger platform ecosystem (platform owner, all developers and all end-users). This, in turn, may be embedded within a larger stakeholder ecosystem. The platform owner should be aware of the relevant ecosystems and the roles and responsibilities within each. A useful approach is to create personas for ecosystem actors to understand their roles, identify their potential for value creation, capture and delivery, relevant financial considerations and adoption mechanisms for each actor involved.

Competition amongst platforms is a dynamic and complex landscape [92]. Four possible sources of competition include (1) the competition between the developers within the platform ecosystem, (2) tensions arising from overlapping functionality between developers and platform, (3) emerging technologies threatening the platform and (4) competing ecosystems. The first two sources arise inside the platform ecosystem, whereas the latter two occur outside the platform ecosystem.

In the SSA health context, a platform owner should be aware of the rules, regulations, protocols and regulatory authorities within the selected ecosystem and direct environment. In the health context, it is particularly important to build trust within the ecosystem. This may require partnering with local and trusted organisations and deliberate trust-building initiatives.

The third part of the platform development dimension relates to the ‘design and governance’. It comprises three general elements and four elements related specifically to SSA health. Firstly, the platform owner should focus on defining the value creation logic. This may include clarifying the platform offering, establishing who will be the value-creating actors within the ecosystem and formulate a monetisation strategy to capture and fairly distribute the value. Secondly, some options for monetisation are included. The platform owner may choose to charge a subscription fee, to determine the price based on the project scope, to charge transaction fees, calculate the charges based on a percentage profit, implement credits for users or offer enhanced access at a greater cost. Other options include charging per user or per feature costs, based on the size of the customer base, licensing agreements, outsourcing certain functionalities or obtaining sponsorships for certain projects.

The third element is specifically design-related. Mindful of the importance of being aware of the rules and regulations of the industry and operational environment, a collection of healthcare-related standards, rules and regulations should be considered and incorporated into the software and hardware during the design process. Popular approaches that surfaced during the literature reviews and evaluation process include designing a minimum viable product (MVP) and adopting the Service Oriented Architecture (SOA) [93] or agile approaches [94]. MVP-design refers to designing the end-product, service or technology to satisfy the most crucial specifications, where after further refining takes place and feedback is incorporated. A platform owner should also select a technology stack (tech-stack) design approach for his front-end and back-end designs [95]. Four SSA health-related elements in this part include interfacing with and using EHRs and EMRs, accessing siloed data, integration and interoperability with relevant systems or software and ensuring security and privacy of all data.

The fourth part of ‘platform development’ refers to the ‘management and operation’ of the platform and ecosystem and comprises of five general elements. A platform owner should clearly devise its market-entry strategy, as each application industry and context may have different requirements for success. In the USA, for example, health-related applications may be enforced by medical schemes or large businesses. A partnership with one of these could hence be the key to market entry. In Uganda, on the other hand, most people will not have access to or cannot afford medical aid and therefore a different strategy will be required. Transactional platforms (relating to economic perspective) also have to consider the chicken-or-egg-dilemma [96], referring to which side of the market to attract first and how to attract them without the other side being leveraged.

A platform owner should carefully consider possible formal and informal control mechanisms [97], the different openness dimensions available and support structures. Openness does not merely refer to the technology itself [50], but also to governance, R&D, general management, marketing and sales and consulting and support. It is imperative that support is provided for the internal platform firm, the developers and end-users.

The final general element of ‘managing and operation’ of the platform and ecosystem relates to the ecosystem health. Five possible components that should be monitored within the ecosystem include the health of the platform owner firm itself, the platform and its software and hardware, the software projects developed on the platform, the environment external to the platform ecosystem and the complex relationships within the platform ecosystem.

Five SSA health-related elements have an impact on the platform development. It is recognised that users may not be aware of or be ill-informed regarding health-issues and the use of technology and may not have access to cutting edge technological devices. They will often have very basic mobile devices and may not be aware of the latest features of smart-devices. In addition, they may also face challenges regarding access mobile data, including availability and cost [69, 70, 98].

These elements all affect the design process and the platform owner/developers need to be aware of the implications. Platform designers should therefore be mindful to design applications so as to reduce data traffic, as uploading and downloading speeds may be limited. In addition to the end-user context, the end-user her/himself should be considered to ensure initial and sustained adoption. Initiatives in SSA involving platforms may not go beyond the pilot stage as a result of decreasing usage or lack of funding.

The fifth and final part of the platform development dimension refers to the ‘evolution of the platform and ecosystem’ and comprises ten considerations for evolution (as indicated in Table 6). A platform owner should be aware of its maturity and adjust its goals and priorities for each stage of its life cycle. Evolution of the platform and ecosystem may include evolving the platform ecosystem, the platform, the platform firm itself or one or more of the platform projects.

**Table 6:**
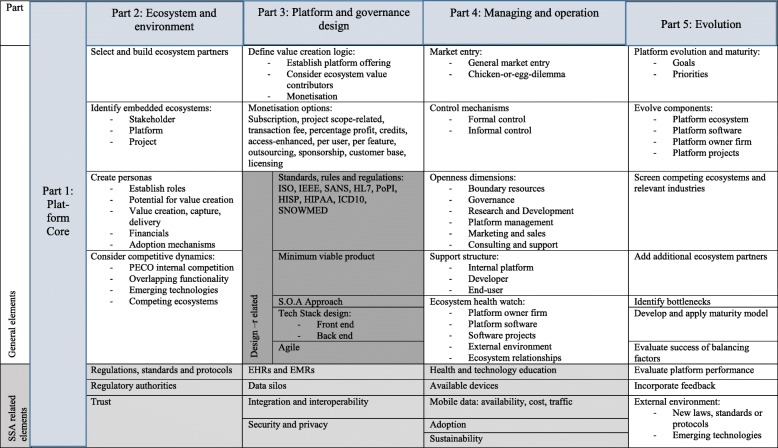
Framework - Dimension two: Platform development

The platform owner should also monitor competing ecosystems and relevant industries for indications of emerging competition or future trends and technologies. As a result, the platform owner may choose to add additional ecosystem partners to build and/or evolve the ecosystem. Additional elements include the identification of bottlenecks that are inhibiting growth, the development and application of a maturity model for continuous improvement, evaluation of the platform performance based on predefined key performance indicators (KPIs), the incorporation of feedback from the ecosystem and its use as sources for further development.

The platform owner can also evaluate the success of balancing factors such as openness and modularity of its platform boundaries. Finally, the external environment should continuously be monitored for the proclamation of new legislation, protocols, standards or technologies.

## Discussion and conclusions

The proposed health-technology platform framework can be used as a tool to inform and aid platform owners in designing, developing and implementing healthcare-related platforms and resulting ecosystems, specifically in the SSA context. The tool was deliberately developed to integrate the economic and engineering platform perspectives and to be usable by different types of platforms. Subsequently, the platform framework is generalised and not all concepts and elements will be relevant for all platforms. The industry case study evaluation stage, confirmed the framework as a useful tool for platform owners.

The framework and associated development tool can be useful in practice as well as a research instrument. Mindful that research frameworks and tools are often not practically usable by industry due to it being complicated and difficult to follow and relate to [81, 99], this tool was developed to be easily understandable and including self-explanatory terms that can be applied to different types of platforms. The framework was also developed with an ecosystem focus, considering each of the ecosystem actors individually before combining their needs and characteristics into the framework [92]. The ecosystem perspective also informs on the different ecosystem actors and functioning in the SSA context, possibly contributing to a better understanding and subsequent adoption of such platforms in this context. Specific challenges that platform owners could face were investigated and incorporated into the framework [19, 100].

The framework supplemented by additional information and insights into the SSA health context obtained from the evaluation stages, lead to the development of a practical management tool. The tool is comprises six canvasses, viz.

- Pre-use canvas
- Overview canvas
- Platform Owner canvas
- Developer canvas
- End-user canvas
- Platform Development canvas

These canvasses provide a practical approach and interpretation of this framework and was proven to be useful to platform owners during its evaluation stages. These canvasses can be accessed in the Addition files section of this article.

A number of insights into the broader SSA health context are also worth mentioning. SSA as a developing area will require different design and management strategies that other developed countries [22]. At the time this research was conducted, little published research dealing with platforms in these contexts was available. Technological, educational, political and health-related implications result in a complex landscape for technology platforms and therefore require increased research. A summary of the SSA health context-related insights is provided in Table 7.

**Table 7:** Summary of SSA health-related elements of framework

SSA related aspects	Motivation/ description
Regulations and standards	Compliance to specific regulations and standards mandatory in health industry.
Regulatory authorities	For example, in SA’s public health sector, the NDoH is a key stakeholder in the ecosystem. Platform owners would need to consider such authorities if they want access to government and public hospitals.
Building trust	Trust may need to be built with users where technology is still unfamiliar. Within local authorities there might also be a sense of scepticism due to several similar initiatives that failed.
EHRs and EMRs	The platform might need access or enable use of existing EHRs or EMRs. These can be non-existent or hard to access in these contexts.
Data silos	There are currently no clear interoperability standards for this context and subsequently there are several silos of data that would require specialised effort to access.
Integration and interoperability	Integration and interoperability with systems in this context may be particularly difficult as they are often outdated and undefined.
Security and privacy	In SA, the Protection of Personal Information (PoPI) Act is a fundamental part of its citizens. Effort should be taken into keeping data, such as HIV statuses, secure and private whilst complying with the Act.
Health and technology education	The end users, particularly in rural areas, may not be digitally literate. These end-users are often uneducated on health-related issues. This affects adoption and sustained use.
Sustainability	Financial sustainability, as well as adoption and sustained use by the end users are particularly challenging in developing environments.
Available devices	Some end users may only have access to very simple and old mobile devices, therefore resulting in limitations and constraints in the design process.
Adoption	As technology may not be familiar for all, adoption might be slow and require support and active change management.
Data availability and cost	In rural areas the end users may have limited connectivity and may not be able to afford mobile data. This has an effect on back-ups, sending and storing of data. Often end-users work with pay-as-you-go data and will reject the app if is it costs anything at all.
Data traffic	Heavy data traffic may prevent apps from working sufficiently, therefore implicating the design.

Whereas the framework can be useful, as was demonstrated in the case study, it will benefit from further improvement. Future work could include further evaluation and refining of the framework, particularly as more experience is gained through its practical use. As proposed, the framework is generalised for different types of platforms, therefore future work could also include refining the elements for each type of platform. The framework concepts are also not yet ranked based on importance or weightings. Some concepts have a higher importance than others and rankings for the different categories in the framework could prove useful in future. The framework can be developed further for different geographical regions and health systems.

The platform framework lends itself to form the basis for a software or app implementation, either as a product or a service. This can be a promising avenue for further research given the demand for software-based products and services and the need to continuously revaluate, adapt and evolve.

### Supplementary information

**Additional file 1.** Pre-use Canvas. This canvas aims to guide the platform owner through establishing the profile for his own platform. Four platform profile factors were found to influence the approach towards the framework: (1) the platform type, (2) whether the platform is an internal or external platform, (3) the platform distribution channels and (4) the application industry of the platform.

**Additional file 2.** Overview Canvas. The Overview Canvas has three functions. Firstly, the focus of the Overview Canvas is to show how these two dimensions overlap and thereby give an overview of the framework content. The Overview Canvas therefore comprises the platform development parts as the rows and the ecosystem actors as the columns. At the intersection of the two dimensions, the canvas includes the relevant categories and subcategories from the Ecosystem Canvasses. These primary and secondary categories highlight important considerations at each respective intersection point. Secondly, the Overview Canvas acts as a reference guide by which the platform owner can navigate through the remainder of the framework. An example is illustrated in Figure 98: if the focus is specifically on the end user (column 3) and the platform and governance design part (row 3), the Overview Canvas can be used to guide the platform owner where to focus his attention within the framework canvasses for more information. Figure 98 indicates the intersection point (dotted red) on the Overview Canvas and how it refers the framework user to the correct ecosystem canvas categories. Thirdly, the Overview Canvas can also be used to understand platform design, development and implementation on a high level. The primary and secondary categories on this canvas were selected to be descriptive in order to provide understanding on a high level. By understanding the two dimensions and their intersection points, the platform owner can potentially develop his own, customised breakdown of these primary and secondary categories. The platform owner therefore does not have to be limited to the category breakdown given in the remainder of the framework. Following this Overview Canvas are the dimension one Ecosystem Canvasses.

**Additional file 3.** Platform Owner Canvas. The Platform Owner Canvas aims to inform a platform owner what to consider regarding his own firm, platform and the ecosystem forming around its platform. The user of the canvas should approach it by putting on the ‘platform owner’s hat’. The Platform Owner Canvas comprises four main categories that have proven to be key in the design, development and implementation processes. The first category refers to the platform owner’s own firm and the design thereof. Within this category, the concepts were grouped according to their respective relations to the platform vision, the internal organisation and the operations within the firm. The platform vision includes concepts concerning the core of the platform, its purpose and future trajectory. The second category comprises the platform design with two subcategories. These two subcategories refer to the technology infrastructure and corresponding rules and regulations. Technology infrastructure specifically includes the technical and software considerations of the platform. Next, the platform ecosystem considerations relating to the platform’s ecosystem and its external environment are included. The external environment focus on competition and it emphasises the need to look outside of the platform and ecosystem for sustained success and evolution. The final category for this canvas includes the evolution of the platform. Subsequent to understanding of the platform owner’s key concepts, the Developer Canvas follows.

**Additional file 4.** Developer Canvas. The platform owner needs to put on the ‘developer hat’ when using the Developer Canvas and understand what developers’ characteristics and needs are regarding the platform and ecosystem as shown in Figure 101. The developers refer to the actors that are developing the extensions or modules such as applications using the platform. As discussed in the Pre-use Canvas, the developers can either be internal or external to the firm. Key focus areas of developers include the platform and ecosystem entry barriers, how well the platform enables them to innovate, the availability of boundary resources, how open these boundaries are and the ability to provide feedback regarding the platform. The Developer Canvas is divided into five main categories that aim to provide a general understanding of what a platform owner should consider with regard to the developers using his platform. The first category refers to the entry barriers. The entry barriers are those factors that would either cause a developer to resist joining the platform or encourage them to join the platform. The entry barriers were categorised according to their relation to the platform technology, to how the platform firm is perceived (mission), how value will be configured within the ecosystem and what the platform ecosystem looks like. Subsequent to the entry barriers, are the general ecosystem considerations. These refer to how the platform owner should manage and govern the developers within the ecosystem. The final three categories refer to the technology, control and support. The technology infrastructure includes what should be enabled or considered regarding the developers. Fourthly, the canvas elaborates on the control the platform owner should have in place. This specifically refers to the rules and regulations and to informal and formal control mechanisms. The final category is developer support. The support provided to developers can be from external developer communities or through the platform and platform firm itself. The common purpose of all these categories is to enable and encourage developers to develop complementary products, services or technologies for end users.

**Additional file 5.** End-user Canvas. The end users portrayed in the canvas comprise two components: (1) a client acting as an intermediary between the platform owner and (2) the actual user of the product, service or technology developed using the platform. The canvas is therefore split according to these two components. In the case of no client being present, the remainder of the canvas can still be used as normal. The focus areas of the client typically include the price of the initiative, how value will be created through it and whether their specifications are being met. The actual users of the products, services or technologies typically focus on its usability in their context, other similar products available, user satisfaction, its sustained adoption and enabling user feedback. The canvas layout includes dedicated sections for both of the client and actual end user respectively. The client component of the canvas is presented first and covers two main categories of interest. The first category refers to the technology requirements. This includes determining the requirements of the product, service or technology as well as its specifications. The second category refers to the suggested plan of action, specifically with regards to the financial considerations, the operation of the product, service or technology and its evolution. The categories for the actual end-user component include the context of use, operation of the product, service or technology and its user interface. Thoroughly investigating the context of use is crucial for success. The platform owner should be informed regarding all deployment-related activities, enabling and incorporating feedback and focus on complying with all privacy and security standards and protocols. These considerations cover the major operational factors with regards to the end users. The final category is the interfaces of the products, services or technologies. Detailed attention should be given to the usability and general design of the front end as this directly influences the success of adoption and the potential subsequent health-related improvements.

**Additional file 6.** Platform Development Canvas. Platform Development Canvas comprises five parts: (1) platform core, (2) ecosystem and environment, (3) platform and governance design, (4) managing and operation and (5) evolution. The Platform Development Canvas’ layout includes the five parts of platform development, additional SA health considerations and relevant literature for each development part. The canvas has three overarching aims. The first aim of this canvas is to facilitate the development of a strategy for the platform design, development and implementation as the canvas guides the platform owner through the typical development parts. Secondly, where the Ecosystem Canvasses educate the platform owner on various topics, the Platform Development Canvas gives structure to their implementation. The final aim of this canvas is to inform on practical and actionable elements that draw from the Ecosystem Canvasses. In other words, it also provides possible interpretations of the dimension one canvasses. The Platform Development Canvas can also be used for software products developed on the software platform.

## Figures and Tables

**Fig. 8:**
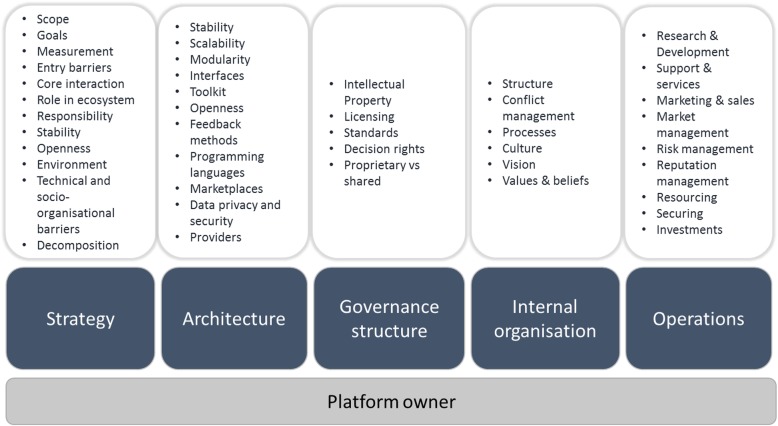
Inventory framework: Platform owner level

**Fig. 9:**
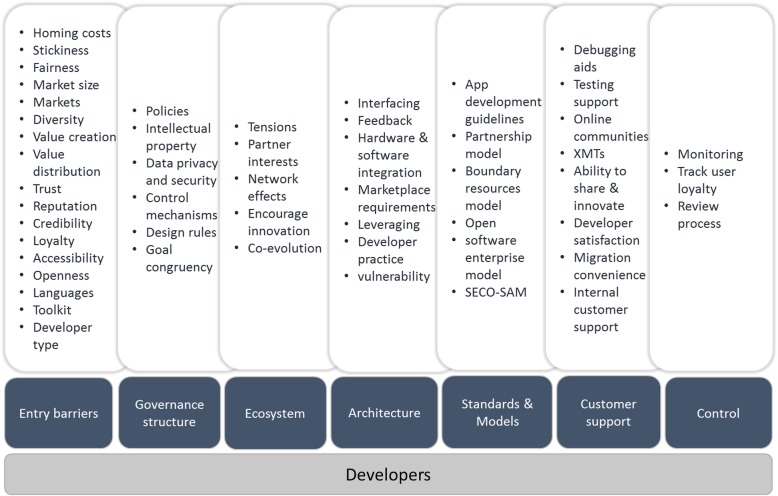
Inventory framework: Developer level

**Fig. 10:**
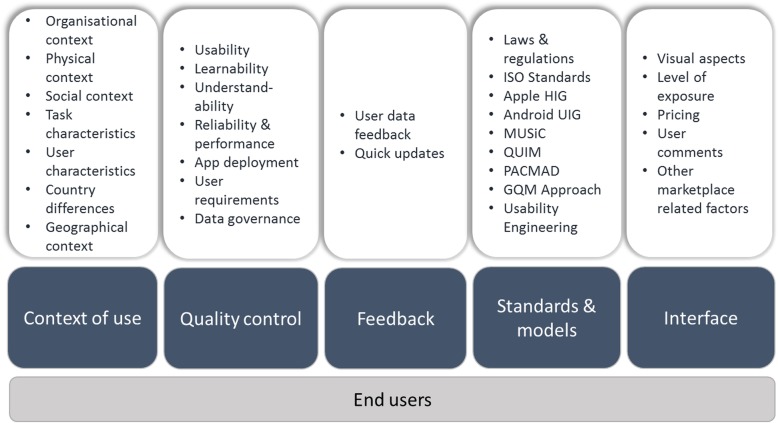
Inventory framework: End-user level

**Fig. 11:**
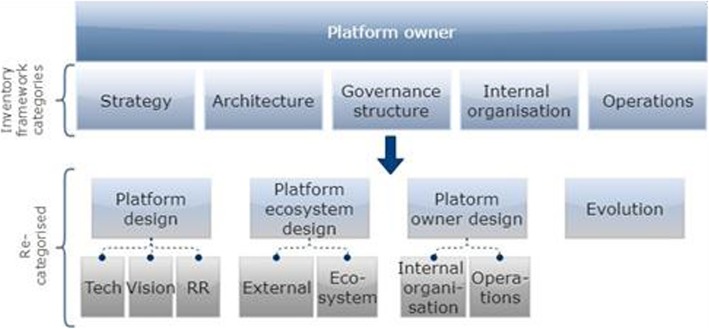
Restructuring of platform owner level categories

**Fig. 12:**
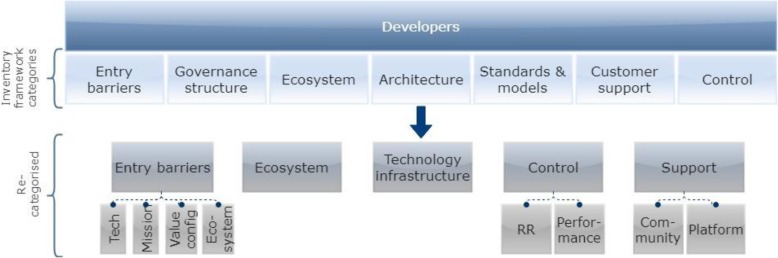
Restructuring of developer level categories

**Fig. 13:**
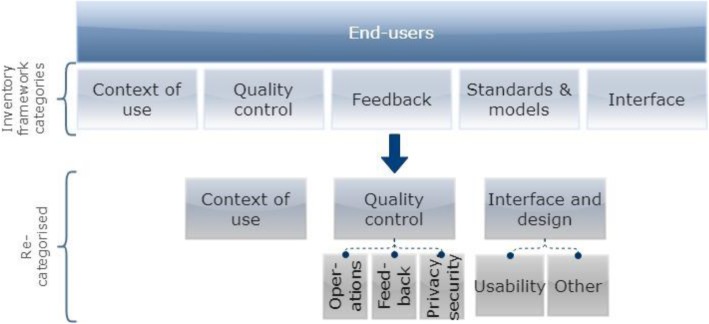
Restructuring of end-user level categories

## Data Availability

The data that support the findings of this study are available from the authors, but restrictions apply to the availability of these data, which were used under license for the current study, and so are not publicly available. Data are however available from the authors upon reasonable request.

## Appendix A

The first level of the inventory framework considers the platform owner. This level comprises five categories: (1) strategy, (2) architecture, (3) governance structure, (4) internal organisation and (5) operations.

## Abbreviations

CEO: Chief Executive Officer
CFA: Conceptual Framework Analysis
EHR: Electronic Health Record
EMR: Electronic Medical Record
HIPAA: Health Insurance Portability and Accountability Act of 1996
HISP: Health Information Systems Programme
HIV: Human Immunodeficiency Virus
HL7: Health Level Seven
ICT: Information and Communications Technology
IoT: Internet of Things
IT: Information technology
KPI: Key Performance Indicator
MS: Microsoft
MVP: Minimum Viable Product
NDoH: National Department of Health
NHI: National Health Insurance
PECO: Platform Ecosystem
PoPI: Protection of Personal Information Act
R&D: Research and Development
SA: South Africa
SECO: Software Ecosystem
SOA: Service Oriented Architecture
SSA: Sub-Saharan Africa
USA: United States of America

## Footnotes

1 Investment platforms consist of companies that have developed a platform portfolio strategy and act as a holding company, active platform investor or both – these type of platforms are not deemed relevant for this study and are therefore not considered further.

2 C1 - Excluding conference reviews, panel discussions and lecture notes.; C2 - Empirical soundness and Academic rigour of paper

3 The construct of this dimension is based on insights gained during the evaluation process, whereas Dimension One draws mainly from literature. Therefore Dimension One is thoroughly referenced from literature, whereas Dimension Two’s development was based on the evaluation of the MomConnect case study, interviews and the industry-based case study.
