# Supplementary material for: The design and development of technology platforms in a developing country healthcare context from an ecosystem perspective
Source: BMC Med Inform Decis Mak. 2020 Mar 12;20:55. doi: 10.1186/s12911-020-1028-0 (PMC7068897; doi:10.1186/s12911-020-1028-0)
Supplement: Supplementary file 1 — Additional file 1. Pre-use Canvas. This canvas aims to guide the platform owner through establishing the profile for his own platform. Four platform profile factors were found to influence the approach towards the framework: (1) the platform type, (2) whether the platform is an internal or external platform, (3) the platform distribution channels and (4) the application industry of the platform. [file 12911_2020_1028_MOESM1_ESM.pdf]

Platform Management Tool
Pre-use Canvas

Establish Platform Profile

Determines perspective and approach towards tool

	Profile consideration	Directing question	Implication(s)
1	Platform Type	Is the platform a transaction platform, an innovation platform or an integrated platform?	This factor will lead to one of the following three cases: (1) Concepts relating to economic view emphasised (2) Concepts relating to engineering view emphasised (3) Concepts relating to both views emphasised
2	Internal or External Platform	Does the platform owner firm function as an internal or external platform firm?	This decision will lead to one of the following two cases: (1) An internal platform will approach ecosystem canvasses with the platform and developers as the same firm (2) An external platform will approach ecosystem canvasses with the platform and developers as separate firms
3	Platform Distribution Channels	How will the platform and end-products be distributed or accessed?	These decisions will emphasise certain concepts and categories. For example: cloud-based, online or offline access or distribution via a marketplace will affect platform design
4	Application Industry	In which industries will the platform or its end products, services or technologies be used?	Certain industries (e.g. health) will require special attention to certain categories and concepts. For example concepts relating to standards, protocols, control mechanisms, context of use, etc.

Tool Overview

Graphical overview of canvasses included in tool

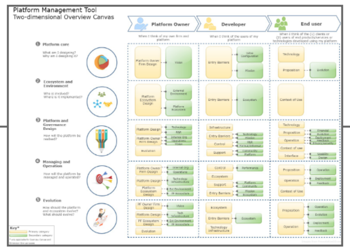

Overview Canvas:

Links two dimensions

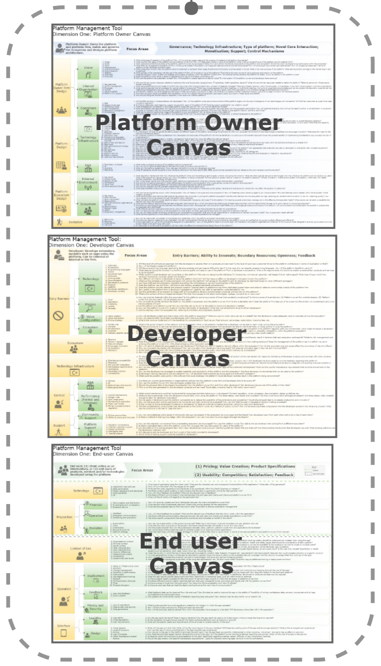

Dimension One: Ecosystem actors

Canvasses' aims:

- Highlight key concepts for consideration
- Ask key questions regarding concepts
- Categorise concepts

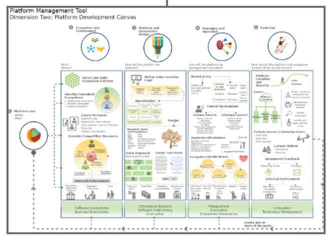

Dimension Two: Platform development

Canvas aims:

- Outlines development categories
- Presents possible interpretations of Ecosystem Canvasses' concepts (tools, methods, etc.)
- Links categories to SA health
