# Supplementary material for: The design and development of technology platforms in a developing country healthcare context from an ecosystem perspective
Source: BMC Med Inform Decis Mak. 2020 Mar 12;20:55. doi: 10.1186/s12911-020-1028-0 (PMC7068897; doi:10.1186/s12911-020-1028-0)
Supplement: Supplementary file 2 — Additional file 2. Overview Canvas. The Overview Canvas has three functions. Firstly, the focus of the Overview Canvas is to show how these two dimensions overlap and thereby give an overview of the framework content. The Overview Canvas therefore comprises the platform development parts as the rows and the ecosystem actors as the columns. At the intersection of the two dimensions, the canvas includes the relevant categories and subcategories from the Ecosystem Canvasses. These primary and secondary categories highlight important considerations at each respective intersection point. Secondly, the Overview Canvas acts as a reference guide by which the platform owner can navigate through the remainder of the framework. An example is illustrated in Figure 98: if the focus is specifically on the end user (column 3) and the platform and governance design part (row 3), the Overview Canvas can be used to guide the platform owner where to focus his attention within the framework canvasses for more information. Figure 98 indicates the intersection point (dotted red) on the Overview Canvas and how it refers the framework user to the correct ecosystem canvas categories. Thirdly, the Overview Canvas can also be used to understand platform design, development and implementation on a high level. The primary and secondary categories on this canvas were selected to be descriptive in order to provide understanding on a high level. By understanding the two dimensions and their intersection points, the platform owner can potentially develop his own, customised breakdown of these primary and secondary categories. The platform owner therefore does not have to be limited to the category breakdown given in the remainder of the framework. Following this Overview Canvas are the dimension one Ecosystem Canvasses. [file 12911_2020_1028_MOESM2_ESM.pdf]

Platform Management Tool
Two-dimensional Overview Canvas

	Platform owner	Developer	End user
	When I think of my own firm and platform	When I think of the users of my platform	When I think of the (1) clients or (2) users of end products/services or technologies developed using my platform
**1 Platform core**<br>What am I designing?<br>Why am I designing it?	Platform Owner Firm Design – Vision	Entry Barriers – Value Configuration<br>Entry Barriers – Mission	Technology<br>Proposition – Evolution
**2 Ecosystem and Environment**<br>Who is involved?<br>Where is it implemented?	Platform Ecosystem Design – External Environment<br>Platform Ecosystem Design – Platform Ecosystem	Entry Barriers – Ecosystem	Context of Use
**3 Platform and Governance Design**<br>How will the platform be realised?	Platform Design – Tech Infrastructure; R&R<br>Platform Owner Firm Design – Internal Org; Operations; Vision<br>Evolution	Technology Infrastructure<br>Entry Barriers – Technology; Mission<br>Control – R&R; Performance<br>Support – Community; Platform	Technology<br>Proposition – Financial; Evolution<br>Operation – Deployment; Feedback; Privacy/security<br>Context of use<br>Interface – Usability; Design
**4 Managing and Operation**<br>How will the platform be managed and operated?	Platform Owner Firm Design – Internal Org; Operations<br>Platform Design – Technology Infrastructure<br>Platform Ecosystem Design – Ext Environment; PF Ecosystem	Control – Performance<br>Ecosystem<br>Support – Platform; Community<br>Entry Barriers – Mission; Ecosystem	Proposition – Operation<br>Operation – Deployment; Feedback<br>Context of Use
**5 Evolution**<br>How should the platform and ecosystem evolve?<br>What should evolve?	PF Owner Firm Design – Vision<br>Platform Design – Technology Infrastructure<br>PF Ecosystem Design – Ext Environment; PF Ecosystem<br>Evolution	Ecosystem<br>Entry Barriers – Ecosystem<br>Technology Infrastructure	Proposition – Operation; Evolution<br>Operation – Deployment; Feedback

Key*
- Primary category
- Secondary category

* only applicable for Overview Canvas and Dimension One canvasses

Images from PresentationGO.com
