# Supplementary figures and images for: The design and development of technology platforms in a developing country healthcare context from an ecosystem perspective
Source: BMC Med Inform Decis Mak. 2020 Mar 12;20:55. doi: 10.1186/s12911-020-1028-0 (PMC7068897; doi:10.1186/s12911-020-1028-0)

# Platform Management Tool

## Dimension One: Platform Owner Canvas

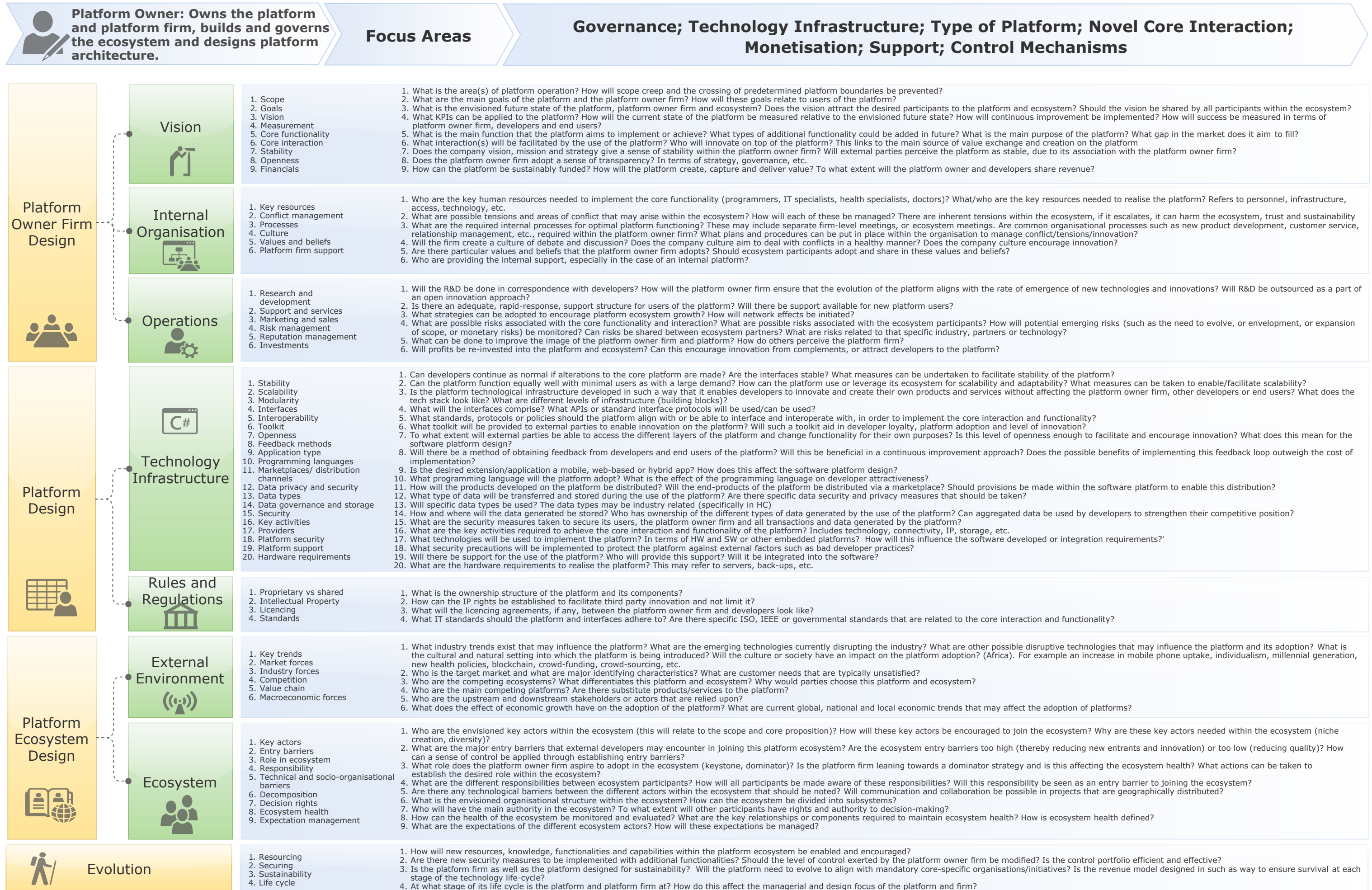

Supplement: Supplementary file 3 — Additional file 3. Platform Owner Canvas. The Platform Owner Canvas aims to inform a platform owner what to consider regarding his own firm, platform and the ecosystem forming around its platform. The user of the canvas should approach it by putting on the ‘platform owner’s hat’. The Platform Owner Canvas comprises four main categories that have proven to be key in the design, development and implementation processes. The first category refers to the platform owner’s own firm and the design thereof. Within this category, the concepts were grouped according to their respective relations to the platform vision, the internal organisation and the operations within the firm. The platform vision includes concepts concerning the core of the platform, its purpose and future trajectory. The second category comprises the platform design with two subcategories. These two subcategories refer to the technology infrastructure and corresponding rules and regulations. Technology infrastructure specifically includes the technical and software considerations of the platform. Next, the platform ecosystem considerations relating to the platform’s ecosystem and its external environment are included. The external environment focus on competition and it emphasises the need to look outside of the platform and ecosystem for sustained success and evolution. The final category for this canvas includes the evolution of the platform. Subsequent to understanding of the platform owner’s key concepts, the Developer Canvas follows. [file 12911_2020_1028_MOESM3_ESM.pdf]

# Platform Management Tool

## Dimension One: End-user Canvas

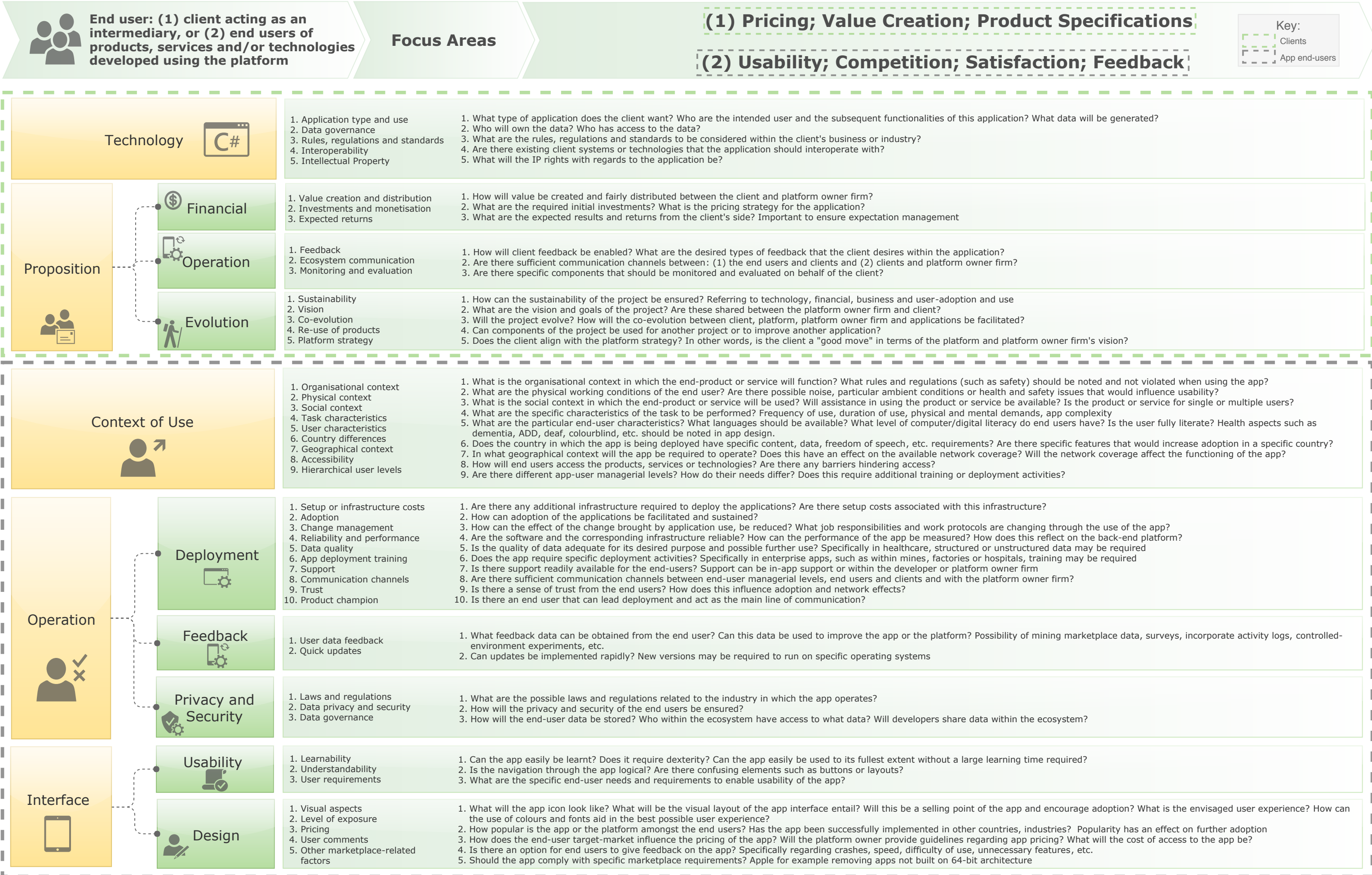

Supplement: Supplementary file 5 — Additional file 5. End-user Canvas. The end users portrayed in the canvas comprise two components: (1) a client acting as an intermediary between the platform owner and (2) the actual user of the product, service or technology developed using the platform. The canvas is therefore split according to these two components. In the case of no client being present, the remainder of the canvas can still be used as normal. The focus areas of the client typically include the price of the initiative, how value will be created through it and whether their specifications are being met. The actual users of the products, services or technologies typically focus on its usability in their context, other similar products available, user satisfaction, its sustained adoption and enabling user feedback. The canvas layout includes dedicated sections for both of the client and actual end user respectively. The client component of the canvas is presented first and covers two main categories of interest. The first category refers to the technology requirements. This includes determining the requirements of the product, service or technology as well as its specifications. The second category refers to the suggested plan of action, specifically with regards to the financial considerations, the operation of the product, service or technology and its evolution. The categories for the actual end-user component include the context of use, operation of the product, service or technology and its user interface. Thoroughly investigating the context of use is crucial for success. The platform owner should be informed regarding all deployment-related activities, enabling and incorporating feedback and focus on complying with all privacy and security standards and protocols. These considerations cover the major operational factors with regards to the end users. The final category is the interfaces of the products, services or technologies. Detailed attention should be given to the usability and genera [file 12911_2020_1028_MOESM5_ESM.pdf]

## Dimension Two: Platform Development Canvas

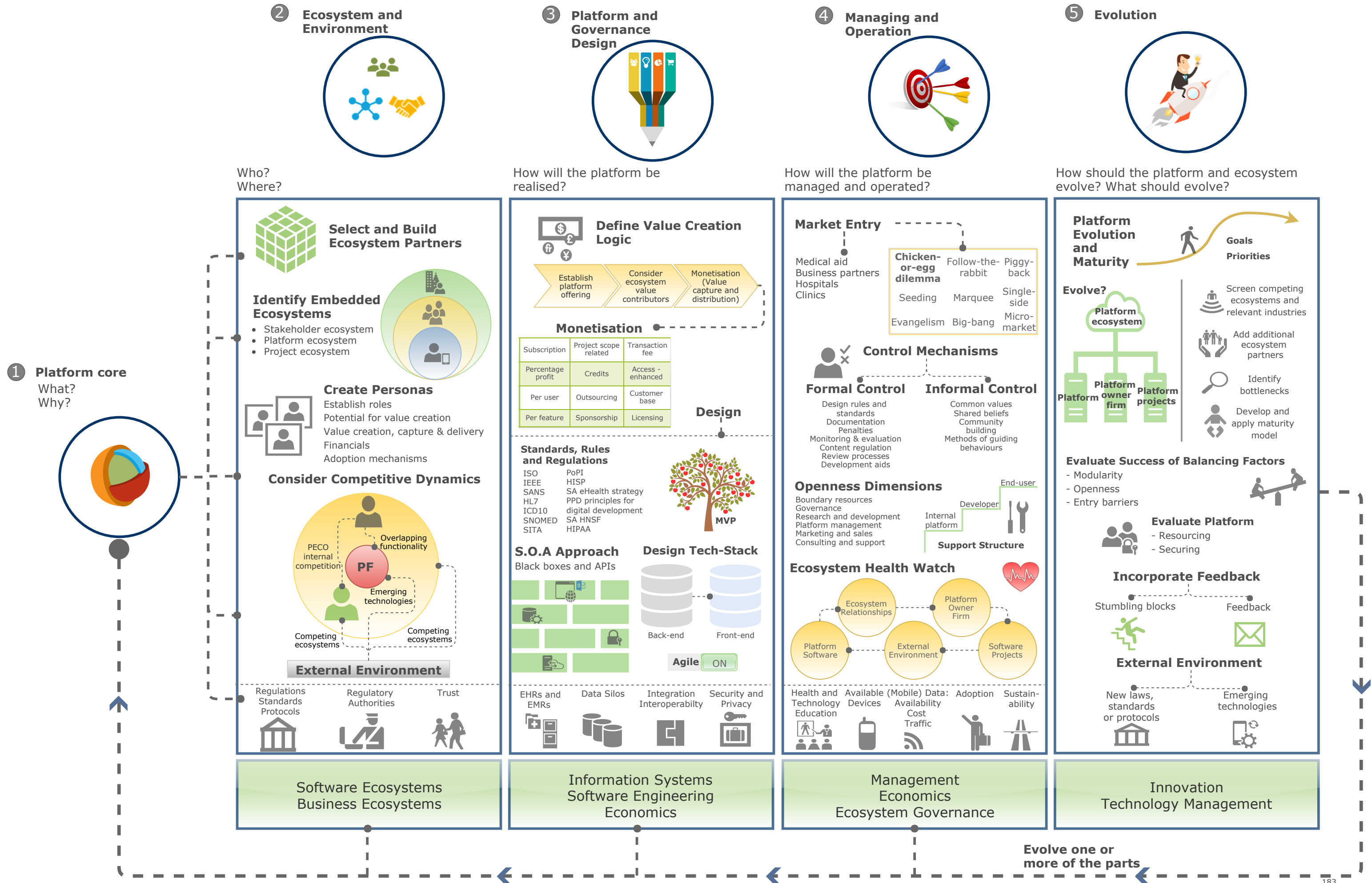

Supplement: Supplementary file 6 — Additional file 6. Platform Development Canvas. Platform Development Canvas comprises five parts: (1) platform core, (2) ecosystem and environment, (3) platform and governance design, (4) managing and operation and (5) evolution. The Platform Development Canvas’ layout includes the five parts of platform development, additional SA health considerations and relevant literature for each development part. The canvas has three overarching aims. The first aim of this canvas is to facilitate the development of a strategy for the platform design, development and implementation as the canvas guides the platform owner through the typical development parts. Secondly, where the Ecosystem Canvasses educate the platform owner on various topics, the Platform Development Canvas gives structure to their implementation. The final aim of this canvas is to inform on practical and actionable elements that draw from the Ecosystem Canvasses. In other words, it also provides possible interpretations of the dimension one canvasses. The Platform Development Canvas can also be used for software products developed on the software platform. [file 12911_2020_1028_MOESM6_ESM.pdf]
