# Supplementary material for: The design and development of technology platforms in a developing country healthcare context from an ecosystem perspective
Source: BMC Med Inform Decis Mak. 2020 Mar 12;20:55. doi: 10.1186/s12911-020-1028-0 (PMC7068897; doi:10.1186/s12911-020-1028-0)
Supplement: Supplementary file 4 — Additional file 4. Developer Canvas. The platform owner needs to put on the ‘developer hat’ when using the Developer Canvas and understand what developers’ characteristics and needs are regarding the platform and ecosystem as shown in Figure 101. The developers refer to the actors that are developing the extensions or modules such as applications using the platform. As discussed in the Pre-use Canvas, the developers can either be internal or external to the firm. Key focus areas of developers include the platform and ecosystem entry barriers, how well the platform enables them to innovate, the availability of boundary resources, how open these boundaries are and the ability to provide feedback regarding the platform. The Developer Canvas is divided into five main categories that aim to provide a general understanding of what a platform owner should consider with regard to the developers using his platform. The first category refers to the entry barriers. The entry barriers are those factors that would either cause a developer to resist joining the platform or encourage them to join the platform. The entry barriers were categorised according to their relation to the platform technology, to how the platform firm is perceived (mission), how value will be configured within the ecosystem and what the platform ecosystem looks like. Subsequent to the entry barriers, are the general ecosystem considerations. These refer to how the platform owner should manage and govern the developers within the ecosystem. The final three categories refer to the technology, control and support. The technology infrastructure includes what should be enabled or considered regarding the developers. Fourthly, the canvas elaborates on the control the platform owner should have in place. This specifically refers to the rules and regulations and to informal and formal control mechanisms. The final category is developer support. The support provided to developers can be from external developer commu [file 12911_2020_1028_MOESM4_ESM.pdf]

Platform Management Tool
Dimension One: Developer Canvas

Developer: Develops extensions, modules such as apps using the platform. Can be external or internal to the firm.

Focus Areas

Entry Barriers; Ability to Innovate; Boundary Resources; Openness; Feedback

Entry Barriers

Technology

1. Openness
2. Accessibility
3. Programming languages
4. Toolkit
5. Developer type
6. Stickiness
7. Homing costs
8. Documentation
9. Standards and protocols
10. Support
11. Usability
12. Developer satisfaction
13. Developer context

1. Will the level of architectural openness limit the developers to develop their own products and services? Is the level of openness a potential threat to the platform architecture in terms of duplication or theft?
2. How will developers access the platform?
3. Will the programming language required to develop products and services be difficult to learn? In the case of a non-popular programming language, why will the platform benefit by using it?
4. What features should be included in a toolkit to ensure quality and ease of use of the platform? From a developer's perspective, what are the requirements of a toolkit to enable complementary products and services to be developed on the platform?
5. What would a typical developer aim to achieve on the platform? What type of app would developers develop (enterprise, commercial, personal, web-based, hybrid, native apps)? Further extensions, modules, added functionality?
6. How difficult will it be for a developer to leave the platform (lock-in)? Will this have an effect on a developer's motivation to join this platform?
7. What are the complete set of costs associated with joining the platform? Will access to the platform and its interfaces be restricted based on costs (different packages)?
8. Are there sufficient documentation available to describe the comprehensive use and functionalities of the platform?
9. Does the platform use and follow well-known and industry accepted standards and protocols?
10. Does the platform provide internal and external support? Internal refers to platform owner firm's dedicated support team and external refers to communities outside of the platform firm
11. How usable is the platform from a developer's perspective? Usability can refer to learnability, understandability, etc.
12. Can the satisfaction of developers be measured? Is it enjoyable to develop complementary products and services on the platform?
13. What is the context of use of the envisaged developers? Do they have access to the latest technologies, laptops, connectivity or resources?

Mission

1. Trust
2. Reputation
3. Credibility
4. Loyalty
5. Fairness

1. How can trust be fostered within the ecosystem? Is the platform conveying a sense of trust (not constantly enveloping)? Is there a sense of trust between (1) platform owner firm and developers, (2) platform owner firm and end users, (3) developers and end users?
2. How do external parties perceive the platform, the platform ecosystem and the platform owner firm? Is this a desirable view?
3. Do the platform and platform owner firm do what they set out to do? Is the platform brand and reputation credible?
4. How can the platform owner firm invest to maintain the loyalty of developers? What are rival ecosystems doing to attract developers?
5. Are the dealings within the ecosystem fair (referring to monetary and proprietary aspects)?

Value Configuration

1. Value creation
2. Value distribution
3. Pricing strategy

1. How will developers create and share value within the platform ecosystem? What are ways in which value can be co-created? Can the developers create adequate value to motivate joining the ecosystem?
2. How will value (profits) be divided and distributed throughout the ecosystem?
3. What is the revenue model and will it be beneficial for complementors? Such as pay fixed amount, percentage, subscription, licencing fees, etc.

Ecosystem

1. Market size
2. Markets
3. Envelopment
4. Diversity
5. Industry specific resistance

1. What are the global footprints of the platform and its ecosystem? What is the potential market that a developer will be able to access when joining the ecosystem?
2. Will the end-products or services be distributed via an application portal/marketplace? Will developers be able to distribute freely on these portals if using the platform?
3. How and when will new functionalities be incorporated into the platform? How will developers be encouraged to join the ecosystem if the platform regularly adds functionalities which might envelope a developer?
4. Are there a healthy variety of developers using the platform? Will this encourage developers to join and share value, or result in unhealthy competition within the platform ecosystem?
5. Are there industry-specific barriers to adoption? For example medical doctors concerned about legal implications when joining a tele-health platform

Ecosystem

1. Tensions
2. Partner interests
3. Network effects
4. Encourage innovation
5. Co-evolution
6. Attraction

1. Could risks taken by the platform owner firm (regarding evolution, financial, scope boundaries) affect the complete ecosystem and thereby result in tensions between ecosystem participants? Relate to risk management and how it is shared by all parties in ecosystem
2. Does the platform owner firm take the best interests of the both themselves and developers into account when making decisions? Does the platform owner firm act in a selfish way at the cost of other ecosystem actors?
3. How will network effects be encouraged amongst developers? Are there any sign of negative network effects within the ecosystem? How do the ecosystem size and scope affect the prevalence of network effects?
4. How can innovation be shared with developers? How can innovation from developers be encouraged? Will external innovation lead to less risk and improved R&D?
5. Is the platform co-evolving with the complete ecosystem? Should ecosystem goals, vision and current markets be re-evaluated?
6. How will planned and potential groups of actors be attracted to join the ecosystem?

Technology Infrastructure

1. Interfacing/compatibility
2. Feedback
3. HW and SW integration
4. Marketplace requirements
5. Leveraging
6. Developer practice
7. Vulnerability

1. To what extent will compatibility with other platforms or systems be enabled or allowed? What level of control will be maintained with regard to interfacing of developer products and services with other systems, solutions or components?
2. Is there a method of obtaining feedback from developers regarding the platform architecture, interfaces, support, etc.? Will developers be encouraged to provide feedback regarding the platform?
3. Do the hardware devices on which the platform and its end-products will be used have an effect on the platform design? Should the platform be able to integrate with existing software systems or components in order to fulfil its function? Should specific data formats or protocols be accommodated?
4. How can the platform owner firm design its platform to enable developers to deliver better apps at their preferred marketplaces? What are the specific marketplace requirements?
5. How can the developers be leveraged to enable scalability and adaptability of the platform and the ecosystem? Are there developer functionalities that can be used by the platform?
6. Is developer training required to use the platform? How will developer best practices be encouraged and enforced if necessary?
7. What could be possible weak points in the platform software that could endanger the platform? Could developer laziness compromise platform security?

Control

Rules and Regulations

1. Policies
2. Intellectual Property
3. Data privacy and security
4. Data governance

1. Are there any current governmental or organisational policies that the platform owner firm and developers should be aware of?
2. How can the platform owner firm help developers protect their own IP?
3. Should the personal data of developers be protected from the platform owner firm and from other developers? Can developers be assured of the safety of their data?
4. Who owns the data generated by the developers' products and services? Could data ownership lead to information asymmetry?

Performance (Formal and Informal)

1. Control mechanisms
2. Design rules
3. Goal congruency
4. Monitoring and evaluation
5. Track user loyalty
6. Review process/content regulation

1. What control mechanisms could be implemented to encourage desirable behaviours in developers? Content regulation, review processes, documentation, design guidelines etc.
2. What are the fundamental rules that developers should obey when using the platform? Are these design rules stable and consistent? Do they avoid favouritism amongst developers? Are these design rules versatile enough to allow for developer innovation?
3. Are the goals of developers and the platform compatible (to reduce the possibility of future tensions and competition)? Could aligned goals lead to increased productivity and innovation?
4. How can the ecosystem and its participants be monitored and their performance evaluated? How can M&E be used in managerial decision-making processes?
5. Is the number of active developers increasing or decreasing? Is there an external competing ecosystem causing users and developers to migrate?
6. Will the complementary products/services be reviewed by the platform owner firm as a method of quality control? Who will be reviewing these components? Are the developers aware of this reviewing process? What are the conditions to be met to "pass the test"?

Support

Community Support

1. Online communities
2. Ability to share and innovate

1. How can connectedness and a sense of community between developers in the ecosystem be encouraged and facilitated? Can developers learn from each other and build on top of each other's products and services?
2. Is there a method of sharing knowledge within the ecosystem? How can innovation be encouraged amongst developers?

Platform Support

1. Migration convenience
2. Internal customer support
3. Design guidelines
4. Debugging aids
5. Testing support

1. How can the migration convenience from a competing ecosystem be supported? How can the platform owner firm add to the convenience when joining from a different ecosystem?
2. Is there a dedicated team within the platform owner firm to supply the required support for users of the platform?
3. Can the platform firm and developers learn from major development tools and user interface guidelines (such as Apple HIG and Android UI guidelines)? Are there existing software frameworks that developers can use? What existing platforms can the developers interface with?
4. Will debugging aids be developed and be a part of the toolkit provided to developers?
5. Which forms of testing support could be supplied to developers?
